# Effectiveness of Suicide Prevention Programmes Among Adolescents and Sociocultural Adaptation of Programmes: A Systematic Review

**DOI:** 10.1111/inm.70038

**Published:** 2025-04-10

**Authors:** Rita Pokharel Poudel, Sheeja Perumbil Pathrose, Diana Jefferies, Lucie M. Ramjan

**Affiliations:** ^1^ School of Nursing and Midwifery Western Sydney University Penrith New South Wales Australia; ^2^ Department of Psychiatric Nursing BP Koirala Institute of Health Sciences Dharan Nepal; ^3^ School of Nursing University of Wollongong Wollongong New South Wales Australia

**Keywords:** adolescent, developing countries, mental health, suicide prevention, systematic review

## Abstract

Suicide is a leading cause of adolescent death and is preventable through school‐based programs. This review aimed to identify available suicide prevention programmes for adolescents, their contextualisation, and effects on suicidal behaviours, help‐seeking, knowledge, attitude and coping. This review was conducted by searching four databases following PRISMA guidelines. Articles published till September 2023 were searched, and the search was re‐run in June 2024. All types of studies conducted among adolescents, outside healthcare facilities and published in English were included. Studies that included adolescents with pre‐existing mental health conditions, gender and sexual minority groups were excluded. Quality was assessed using Joanna Briggs Institute tools. The review protocol was registered in PROSPERO (CRD42023469637). A total of 53 of 3663 identified articles were included. There were 41 different suicide prevention programmes used across the 53 studies. Data extraction focused on author, published year, country, study aims, population, interventions, comparison groups, contextualisation and the outcomes of interventions. Most of the studies (64.2%) were conducted in the United States of America. Three studies mentioned the contextualisation of the programme without details. Of the studies measuring suicidal behaviours (82.1%), help‐seeking (66.7%), knowledge (93.8%), attitude (81.8%) and coping (50%), all reported on the effectiveness of programmes. Studies from low‐ and middle‐income countries could not be identified, and very few studies mentioned the contextualisation of programmes. The heterogeneity of the studies and diversity of the programmes, tools, standards of delivery and follow‐up times across the studies made it difficult to draw conclusions about the overall effectiveness of programmes.

## 1 Introduction

Suicide, according to the World Health Organization (WHO), is the fourth leading cause of death among the 15–19‐year age group globally (World Health Organization [WHO] 2021). However, in the United States of America, suicide is listed as the second leading cause of death among young people aged between 10 and 14 years (Centers for Diseases Control and Prevention 2023) and in Europe, there is a reported incidence of adolescent suicide ranging from 4.1% to 23.5%. The wide variability is influenced by a complex interplay of socioeconomic and intercultural differences across countries and specific risk factors (Michael 2012). In low‐ and middle‐income countries (LMICs), the prevalence of suicide is reported from data collected from 61 LMICs, showing that suicide rates ranged from 2.3% to 20.3% among adolescents aged 12–15 years (Dema et al. 2019).

Suicide prevention is prioritised by WHO and is included as an indicator in the United Nations Sustainable Developmental Goals draft fourteenth general programme of work, 2025–2028 (WHO, 3 May, 2024). An initial adolescent suicide screening and prevention programme can be implemented in a school environment and is an opportunity to assess the effectiveness of the programme in a natural setting (Nolta 2014; Pumariega 2021). Previous systematic reviews and meta‐analyses have evaluated school‐based suicide prevention programmes (Kalafat 2003; Miller et al. 2009; Cooper et al. 2011; Cusimano and Sameem 2011; Katz et al. 2013; Robinson et al. 2013, 2018; Baptista et al. 2022; Liljedahl et al. 2023). However, many of these reviews have not presented studies conducted in LMICs nor discussed the contextualisation of programmes when a prevention programme is used in a country that did not develop the programme. Although several studies recommend tailoring programmes before implementing them in different cultures or contexts as the acceptability of the suicide prevention approach may differ from one culture to another (Kalafat and Ryerson 1999; Lakshmi 2016; Liljedahl et al. 2023). Contextualisation involves gaining an understanding of the unique cultural nuances that inform how a programme should be designed and implemented to ensure it is both acceptable and culturally sensitive. This includes appreciating the cultural perspectives of the local community in which the programme will be implemented (Ongeri et al. 2023).

Considering the evidence gap, this systematic review had three specific objectives. First, to explore the available suicide prevention programmes for adolescents 10–19 years of age. Second, to identify how the available programmes have been contextualised for different cultural and geographical contexts. And third, to identify the effects of those programmes on suicide and help‐seeking behaviours, knowledge, attitudes and coping with suicidal thoughts among adolescents.

## 2 Methods

The Preferred Reporting Items for Systematic Reviews and Meta‐Analysis Protocols (PRISMA‐P) recommendations have been used for preparing and reporting this systematic review (Page et al. 2021). The review protocol was registered in PROSPERO (CRD42023469637).

### 2.1 Identification of Studies and Search Strategies

For this systematic review, studies were eligible if they reported suicide prevention programmes among adolescents between the ages of 10–19 years, the age range that defined an adolescent according to WHO (WHO 2021). The review included all types of study designs published in English, and no restrictions were placed on the publication date. This review excluded studies reporting suicide prevention programmes among adolescents in hospitals or healthcare facilities, adolescents with psychiatric problems and gender and sexual minority groups. In addition, the review excluded systematic reviews.

**FIGURE 1:**
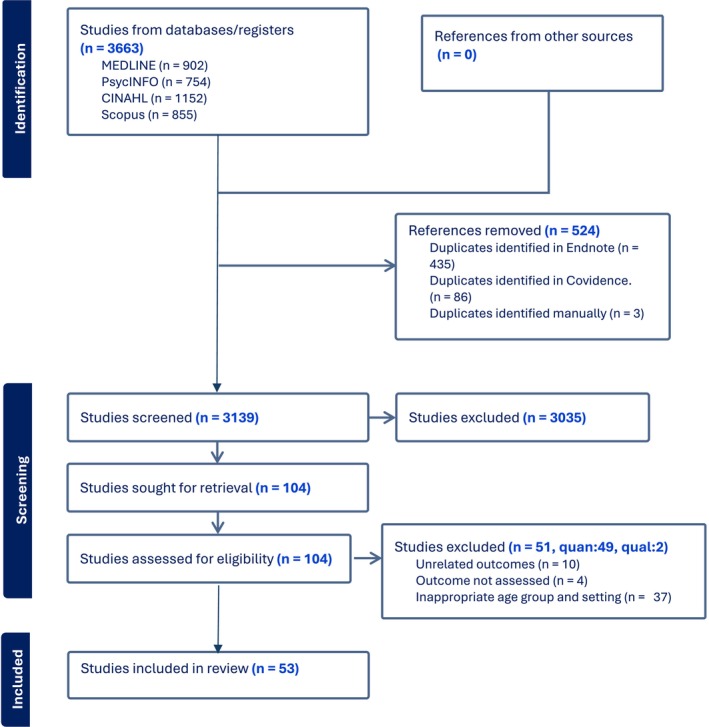
PRISMA flow chart of the effectiveness of suicide prevention interventions for adolescents.

The search was conducted in CINAHL, PsycINFO, Scopus, and Medline databases with a specialist librarian from inception until September 2023 and re‐run in June 2024. The initial key terms were ‘Suicide Prevention Programs’, ‘Adolescents’, ‘Adolescence’ using Boolean operators, Medical Subject Heading (MeSH) terms and truncation. A detailed search strategy is presented in Appendix S1. Following the database search, articles were imported into EndNote version 20. The articles were screened using Covidence, and the Preferred Reporting Items for Systematic Reviews and Meta‐Analyses (PRISMA) guidelines were followed (Page et al. 2021). Details regarding the number of studies retrieved from each database are presented in Figure 1.

### 2.2 Quality Assessment

The critical appraisal tools developed by Joanna Briggs Institute (JBI) were used for quality assessment of the articles (JBI). All experimental, quasi‐experimental and cohort studies were assessed using 13‐item, 9‐item and 11‐item tools respectively. For cross‐sectional and qualitative studies, 8‐item and 10‐item tools for evaluation were used. The quantitative and qualitative research in mixed‐method studies was evaluated separately for quality. All studies were independently assessed by two authors (RP and LR). Conflict in assessment was resolved by a third reviewer (SP or DJ). Terms such as ‘No’, ‘Unclear’, ‘Not applicable’ and ‘Yes’ were assigned to each criterion and each was rated a score of 0, 0, 1 and 1, respectively, with a final percentage calculated. The scores for all included studies are presented in Appendix S2.

### 2.3 Data Extraction

Following the screening and quality assessment, studies were grouped according to design. A custom template was developed based on PICO (Population, Intervention, Comparison, Outcome) for data extraction. Data were extracted independently by two authors (RP and LR) and conflict was resolved by a third author (SP or DJ). Study findings are reported according to PRISMA guidelines (Page et al. 2021).

## 3 Results

A total of 3663 studies were retrieved from all the databases, and 524 duplicates were removed. Following an initial screening of the title, 2199 studies were excluded, and a further 836 studies were removed when the abstracts were screened. The remaining 104 studies were assessed in full for eligibility, and a further 51 were excluded. The reasons for exclusion were unrelated outcomes (*n* = 10), inappropriate age group and settings (*n* = 37), and articles that did not assess outcomes (*n* = 4) (Appendix S3). A final 53 studies met the inclusion criteria and were included in the data extraction (Table 1). One study (Orlins 2023), a thesis, published separate quantitative and qualitative papers, so data were extracted separately (*n* = 54 for data extraction).

**TABLE 1:** Description of the studies.

Author, year, country	Study design	Intervention programme	Aims (to assess)	Tools used	Population (Age in years/grade)	Comparator
Bockhoff et al. (2023), Germany	Two‐factor experimental design	Suicide prevention workshops	Benefits of the programme on suicide prevention	A sociodemographic questionnaire, multiple‐choice question to measure help‐seeking, and helping behaviour. Suicidal Behaviour Questionnaire Revised (SBQ‐R)	NA/8–10	Waitlist control group
Calear et al. (2021), Australia	Cluster‐Randomised Controlled Trial	Silence is deadly	Effects of the programme in help‐seeking, confidence in helping, and help‐seeking stigma for suicide	Sociodemographic questions adapted version of the General Help‐Seeking Questionnaire (GHSQ), an adapted version of the Actual Help‐Seeking Questionnaire (AHSQ), Self‐Stigma of Seeking Help scale, and the Distress Questionnaire‐5	16–18/11–12	Waitlist control group
Wyman et al. (2010), USA	Cluster‐ Randomised Waitlist‐Controlled Trial	Source of Strength	Effectiveness of programme in enhancing protective factors and reducing suicide	Help for Suicidal Peers, Reject Codes of Silence, Maladaptive Coping, Help‐Seeking from Adults at School, and sources of Strength in Coping with Suicidal ideation	M‐15.9/NA	Waitlist control group
Calear et al. (2022), Australia	Cluster‐Randomised Controlled Trial	Source of Strength	Effectiveness of the programme in increasing help‐seeking	Demographics, Adapted versions of GHSQ and AHSQ, Self‐report open‐ended survey	11–17/7–11	Waitlist control group
Pickering et al. (2022), USA	Cluster‐Randomised waitlist‐Controlled Trial	Source of Strength	Programme in reducing suicidal ideation, attempts, knowledge, and attitudes towards suicide while delivering the programme by peer leaders	Demographics, YRBS, and Diffusion of the Source of Strength was assessed in four different dichotomous modalities corresponding to levels of engagement: (1) awareness of, (2) communication about, and (3) active participation in the intervention	M‐15.7/9–12	Waitlist control group
Petrova et al. (2015) USA	Blocked Randomised Posttest only design	Source of Strength	Impact of peer‐delivered positive‐themed messages in reducing suicidal behaviours, identification of trusted adults, and attitude towards suicide	Help Seeking from Adults at School, Reject Codes of Silence, Maladaptive Coping, and Sources of Strength Coping	NA/9–12	Control group
McGillivray et al. (2021), Australia	Cluster‐randomised Trial	Youth Aware Mental Health (YAM)	Impact of the programme on suicidal ideation, self‐reported suicide attempts, literacy, stigma and help seeking	Paykel's Suicidal Feelings in General Population Questionnaire, Literacy of Suicide Scale (LOSS), Stigma of Suicide Scale (SOSS), Patient Health Questionnaire Depression Scale (PHQ‐8), adapted GHSQ and AHSQ	13–16/NA	No comparator
Ciffone (2007), USA	Randomised Controlled Trial	SESH (South Elgin High School) suicide prevention programme	Effectiveness of the programme in changing attitudes about suicide and its effectiveness when delivered by different individuals	An 8‐itemed SEHS survey questions in yes/no format was used to assess the attitude change	NA/10	Waitlist control group
Cigularov et al. (2008), USA	Randomised Controlled Trial	RAPP (Raising Awareness of Personal Power)	Effectiveness of the programme in knowledge and attitudes of suicide, its prevention and self‐efficacy in seeking and offering help for suicidal thoughts	A self‐constructed knowledge, attitude and self‐efficacy assessment tools, adapted from the life satisfaction scale and Esteem Scale (SES)	M‐15.17/NA	Control group
Portzky and van Heeringen (2006), Belgium	Solomon four‐group design	Psychoeducational prevention programme	Effectiveness of the programme on knowledge, attitudes and coping skills for suicidal feelings	Sociodemographic data, the Dutch version of Suicide Information test, Personal experience with suicide, Attitudes towards suicide, A visual analogue scale, Utrecht Coping List, and Beck's Hopelessness Scale (BHS)	14–18/NA	Control group
Kalafat and Elias (1994), USA	Solomon four‐group design	Suicide Awareness Curriculum	Efficacy of curriculum on youth suicidal behaviour, knowledge and attitude towards suicide	Self‐developed agreement tool to assess knowledge. One open‐ended item asked about warning signs of suicide, attitudes towards suicide, help‐seeking and talking about suicide in class were measured by 14‐item true false statements using a 4‐point agreement scale	NA/10	Physical Exercise (PE)
Hart et al. (2020), Australia	Cluster‐randomised controlled trial	Teen Mental Health First Aid (tMFHA)	Effectiveness of programme in improving peer support for adolescents at risk for suicide	The student questionnaire included items adapted from Australian National Survey of Youth Mental Health Literacy and Delphi experts' consensus based on the tMFHA course	15–17/10	Physical First Aid (PFA)
Aseltine Jr. et al. (2007), USA	Randomised Controlled Trial Posttest only design	Signs of Suicide (SOS)	Effectiveness of programme in reducing suicidal behaviour, increasing knowledge and improving attitude	Youth Risk Behaviour Survey (YRBS), Knowledge of depression and suicide was measured with 10 true/false items that reflect the central themes of the SOS programme	NA/9	Control group
Aseltine Jr. and DeMartino (2004), USA	Randomised Controlled Trial Posttest only design	SOS	Effectiveness of programme in reducing suicidal behaviour, increasing knowledge and improving attitude	Same as above	NA/9	Control group
Aseltine Jr. (2003), USA	Quasi‐Experimental Study	SOS	Efficacy of the SOS programme on help seeking for suicidal behaviour	Self‐designed structured questionnaire	NA/high school	No Comparator
Schilling et al. (2014), USA	Cluster‐Randomised Trial	SOS	Effectiveness of SOS in reducing suicidal behaviour, improving knowledge and attitudes, and help seeking or offering	Sociodemographic information adapted version of CDC YBRS for suicide. Knowledge of depression/suicide was measured by a seven true/false items scale that reflected the themes of the SOS programme. A 10‐item summary scale was used to assess attitudes	NA/5–8	Control group
Schilling et al. (2016), USA	Randomised Controlled Trial	SOS	Effects of SOS on self‐reported suicide attempts and suicide behaviours	Sociodemographic information, adapted version of CDC YBRS for suicide, adapted version of knowledge and attitudes about depression and suicide used in other evaluation studies	NA/9	Waitlist control group
Klimes‐Dougan et al. (2009), USA	Randomised Trial	Public service announcement	Possible benefits and untoward effects of programme on depression, suicidal behaviours and coping	Sociodemographic information, Suicide Awareness Questionnaire, help‐seeking and maladaptive‐coping scale	M‐15.24/10–12	Control group
Hooven et al. (2010), USA	Repeated measures randomised design	Counsellor CARE (C‐CARE) and Parent CARE (P‐CARE)	Long‐term effectiveness of programmes in suicidal risk behaviours of adolescents	The High School Questionnaire (HSQ)	M‐15.9/9–12	Minimal intervention control group
Hooven et al. (2012), USA	Repeated measures randomised design	C‐CARE and P‐CARE	Effectiveness of augmenting an intervention with a brief home‐based parent programme in reducing the suicidal risk	HSQ consists of three constructs: Suicide Risk Behaviours, Related Risk and Protective Factors	14–19/NA	Intervention as usual
Thompson et al. (2001), USA	Three‐group repeated measures randomised prevention trial	C‐CARE, CAST (Coping and Support Training)	Efficacy of two interventions for reducing suicidal behaviours and for enhancing protective factors	Same as above	14–19/NA	Control group
Eggert et al. (2002), USA	Three‐group, repeated measures, randomised intervention trial	Counsellor CAST (C‐CAST), CAST	Efficacy of programmes in reducing the suicidal risk	Sociodemographic information and HSQ was used to collect data at 4 time points	14–19/NA	Usual care‐group
Klim‐Conforti et al. (2021), Canada	Randomised Controlled Trial	Harry Potter‐based Mental health literacy programme	Impact of programme on suicidality	Sociodemographic information, Life Problems Inventory (LPI); Revised Child Anxiety and Depression Scale (RCADS)	11–14/7–8	Waitlist control group
Wasserman et al. (2015), European countries	Cluster‐ Randomised Controlled Trial	YAM and ProfScreen	Efficacy of interventions and compare the effectiveness between programmes for suicidal behaviours	Self‐report survey (Demographics), Strengths and Difficulties Questionnaire, Paykel Hierarchical Suicidal Ladder	14–16/NA	Control group
Thompson et al. (2000), USA	Three‐group, repeated measures design	PGC (personal growth class)	Effectiveness of the programme in reducing suicidal risk behaviours	Sociodemographic data, HSQ, A 14‐itemed Teacher support measure, and a 14‐itemed peer support measure	M‐15.86/9–12	Control group
Vieland et al. (1991), USA	Randomised Controlled Trial	Suicide prevention curricula	Impact of programme on suicide and help‐seeking 18 months after delivery	A self‐constructed questionnaire	M‐15.8/NA	Control group
Orbach and Bar‐Joseph (1993), Israel	Randomised Controlled Trial	Experiential suicide prevention programme	Effectiveness of programme in reducing suicidal tendency and improving coping	Sociodemographic information, Israeli Index of Potential Suicide (IIPS), Adolescent's Ego Identity Scale, BHS and Self‐Control Schedule	NA/Junior high school	Control group
Silverstone et al. (2015), Canada	Cohort Study	EMPATHY^a^	Piloting of a novel school‐based approach to reduce depression and suicidality in youth	PHQ‐A, and Hospital Anxiety and Depression Scale (HADS)	11–18/6–12	Universal CBT and OVK
Silverstone et al. (2017), Canada	Cohort Study	EMPATHY	To reduce suicidal thinking in preteens, adolescents and youth students	PHQ‐A, HADS, CRAFFT, Rosenberg self‐esteem scale and Kids screen	11–18/6–12	No comparator
Paschall and Bersamin (2018), USA	Cross‐Sectional	Oregon Healthy Teens (OHT) Survey	Decrease in the likelihood of depressive episodes and suicide risk	Questions based on YRBS	NA/8–11	Not applicable
Wasserman et al. (2012), European countries	Cross‐Sectional	SEYLE^b^	Generating recommendations and enhancing the future potential of such suicide prevention strategies	Open‐ended evaluation questionnaire designed for this study	14–16/NA	Not applicable
Antonio et al. (2020), USA	Qualitative study	HCCI^c^	The impacts of participating in HCCI for suicide prevention	Semi‐structured interview guide was developed for youth leader groups	13–18/NA	Not applicable
White and Morris (2010), Canada	Case study	Classroom‐based suicide prevention programme	Discursive oriented, constructionist investigation into classroom‐based suicide prevention education practices	Classroom sessions and in‐depth interviews were recorded and transcribed	13–15/8–12	Not applicable
Orlins (2023), USA	Nonrandomised waitlist control trial	LifeAct	Effectiveness of the programme in knowledge and help‐seeking intentions	Survey instrument with five parts was constructed and used	13–18/9	No comparator
Orlins (2023), USA	Qualitative thematic analysis		Perception of suicide among teenagers, to inform existing and future suicide prevention programmes and to collect feedback on the programme			
Langdon et al. (2016), USA	Nonrandomised posttest only mixed‐method study	Lumbee Rite of Passage (LROP)	Effectiveness of the model among American Indian youth	Focus groups and evaluation surveys	11–18/NA	No comparator
Le and Gobert (2015), USA	Pre–posttest mixed‐method design	Mindfulness‐based suicide prevention programme	Effectiveness of culturally adapted intervention in preventing suicide of Native American youth	Mindfulness, Healthy Self‐Regulation, Teen Conflict survey and Patient Health Questionnaire (PHQ‐9)	15–20/NA	No comparator
Bailey et al. (2017), Australia	Quasi‐Experimental Study, Pre–posttest evaluation	SafeTALK	Efficacy of programme on suicidal ideation, knowledge, confidence and willingness to talk about suicide, offer and seek help, and distress	Self‐constructed five‐point Likert scale, modified Profile of Mood States (POMS‐M), Adapted Columbia Suicide Scale, and a 4‐point scale of current suicidal ideation	16–18/11–12	No comparator
Kinchin et al. (2019), Australia	Quasi‐Experimental Mixed Method Study	SafeTALK	Effects of programme on suicide‐related outcomes	A mixed‐method questionnaire, and all as above	15–16/NA	No Comparator
Shaffer et al. (1991), USA	Quasi‐Experimental Study	Curriculum‐based suicide prevention programme	Effectiveness of curriculum in increasing knowledge	A 48‐item developed questionnaire	M‐14.8/9–10	Control group
Wise (2023), USA	Quasi‐Experimental Study	E‐CPR (Emotionally connect, Partner, Respond)	Efficacy of the programme in improving confidence in responding to a peer crisis identifying crisis intervention skills	Self‐developed 10‐item survey based on a five‐point Likert scale	14–18/NA	Control group
White (2015), USA	Quasi‐Experimental pre‐ and posttest study	Erika's Lighthouse and Understanding teen depression	Effectiveness of programme in changing adolescents' self‐reported attitude towards suicide	A 14‐item, five‐point Likert scale designed by Erika's Lighthouse based on current programme	NA/High school	No comparator
King et al. (2011), USA	Pre–posttest design	Surviving the Teens	Impact of programme on help‐seeking behaviour	Self‐developed and tested survey tool	14–18/9–12	No Comparator
Strunk et al. (2014), USA	Nonrandomised control group design	Surviving the Teens	Efficacy of programme on improving suicide‐related knowledge and skills	Suicide Intervention Response Inventory (SIRI) and Suicide Intervention Training Assessment (SITA)	13–18/NA	Control group
Pasco et al. (2012), USA	Quasi‐Experimental Study	Campus Connect Training	Efficacy of programme in help‐seeking behaviour	Youth demographic characteristics, modified YRBS. Adapted Service Assessment for Children and Adolescents (SACA) for help‐seeking	M‐19/NA	Short training
Freedenthal (2010), USA	Pre–postintervention survey design	Yellow Ribbon	Effectiveness of programme on suicidal behaviour	A questionnaire was designed and used	M‐15.8/9–12	No comparator
Flynn et al. (2016), USA	Quasi‐Experimental Study	Yellow Ribbon	Effectiveness of programme on knowledge and attitude towards suicide	The Curriculum Assessment Instrument—18 item instrument	11–18/NA	No COMPARATOR
Ciffone (1993), USA	Pre‐post intervention survey design	Sophomore‐level health class	Effectiveness of the programme in reducing suicidal risk and improving help seeking	A shortened version of the Japanese edition of Harter's Perceived competence scale for children	NA/High school	Waitlist control group
Nelson (1987), USA	Pre‐post intervention survey design	Youth Suicide Prevention	Effects of the programme on suicidal ideation among secondary students	Suicidal ideation Questionnaire, Junior Version (SIQ‐JR), Adult Suicidal ideation Questionnaire (ASIQ), Children's Depression Rating Scale (CDRSR), Raynolds Adolescent Depression Scale Version 2 (RADS), and (BHS)	M‐15.5/9–12	No comparator
Ogawa et al. (2022), Japan	Nonrandomised waitlist trial	SOS education output	Effects of the programme on suicidal behaviour, self‐efficacy in prevention and intervention and help‐seeking for suicide	Self‐developed and tested survey tool	14/NA	Waitlist control group
Robinson et al. (2016), Australia	Nonrandomised pre–posttest pilot study	Reframe IT	Effects of the programme on suicide attitudes and knowledge	LifeSavers Attitude and Knowledge Scale (LAKS), Rosenberg Self‐Esteem Scale (RSE) and Self‐Acceptance Scale (SCS)	14–18/NA	No Comparator
Walker et al. (2009), USA	Quasi‐Experimental Study	LifeSavers	Self‐reported changes in knowledge and comfort in communicating suicide	A four‐point Likert scale created by Yellow Ribbon Suicide Prevention Foundation	NA/9–12	No comparator
Nasution et al. (2019), Indonesia	Quasi‐Experimental pre–posttest design	CBT and peer leadership	Effects of the programme on suicidal ideation of adolescents	Beck Scale of Suicidal ideation (BSSI)	NA/11	TKN only control group
Baggio et al. (2022), Switzerland	Nonrandomised cluster‐controlled trial	Stop Suicide	Efficacy of the programme on knowledge of suicide, coping skills and help seeking	Sociodemographic information, French Version of LOSS, self‐developed perceived knowledge of help‐seeking resources, French version of Kessler Psychological Distress Scale (K‐6), French Version of COPE inventory and French version of Columbia Suicide Severity Rating Scale	M‐15.2/NA	Control group

^a^ Empowering a Multimodal Pathway Towards Healthy Youth.

^b^ The Saving and Empowering Young Lives in Europe.

^c^ The Hawai‘i's Caring Communities Initiative.

### 3.1 Characteristics of Studies

Studies were published from 1991 to 2023. Of the 54 studies included, 26 (48.1%) were experimental, 21 (38.9%) quasi‐experimental, 4 (7.4%) were observational, and 3 (5.6%) were qualitative (Table 1). Studies were primarily conducted in high‐income countries across several continents, such as the United States of America (USA) (*n* = 34, 64.2%) followed by Australia (*n* = 7, 13.2%), Europe (*n* = 5, 9.4%) and Canada (*n* = 4, 7.5%), with only three in Asia in upper‐middle [Israel, Indonesia] or high‐income countries [Japan] (*n* = 3, 5.7%) (Orbach and Bar‐Joseph 1993; Nasution et al. 2019; Ogawa et al. 2022). Studies conducted in LMICs and South Asia could not be retrieved. Participants' ages ranged from 11 to 19 years, and the reported mean age range was 13.3 to 19 years (Table 1). Sample sizes across the studies ranged from eight participants in a mixed‐method study reporting on a culturally adapted mindfulness‐based suicide prevention programme for Native American youth (Le and Gobert 2015) to 11 110 participants in the multicentred cluster‐randomised trial (Wasserman et al. 2015). The retention rate ranged from 44% to 100%, and the follow‐up period ranged from immediately to 18 months after the intervention (Appendix S4).

### 3.2 Suicide Prevention Programme Characteristics

There were 41 different programmes implemented across the 53 studies (Table 2). The most frequently used programme was Signs of Suicide (SOS, *n* = 5) (Aseltine Jr. 2003; Aseltine Jr. and DeMartino 2004; Aseltine Jr. et al. 2007; Schilling et al. 2014; Robinson et al. 2016), followed by Source of Strength (*n* = 4) (Wyman et al. 2010; Petrova et al. 2015; Calear et al. 2022; Pickering et al. 2022), Surviving the Teens (*n* = 2) (King et al. 2011; Strunk et al. 2014), SafeTALK (*n* = 2) (Bailey et al. 2017; Kinchin et al. 2019), Yellow Ribbon (*n* = 2) (Freedenthal 2010; Flynn et al. 2016) and EMPATHY (*n* = 2) (Silverstone et al. 2015, 2017).

**TABLE 2:** Description of suicide prevention programmes and their contextualisation.

SN	Programme name	Description of the programme for intervention	Contextualisation
1.	Suicide prevention workshops	A 4‐h training session and psychoeducational intervention, based on Action, Care, Tell (ACT) principles. It was focused on knowledge about suicide and psychological disorders, identification of warning signs and ways to deal with those signs and developing coping strategies	Developed and used in the same country
2.	Silence is deadly	It is a male‐tailored Source of Strength in suicide prevention intervention. The masculine norms that can hinder early, and effective help‐seeking are focused on in this programme	Contextualised for use among males
3.	Source of Strength Program (Wyman et al. 2010; Petrova et al. 2015; Calear et al. 2022; Pickering et al. 2022) (*n* = 4)	A 1‐h orientation to the intervention was provided to school staff. Peer leader training consisted of 4 h of interactive training for peer leaders and adult advisors led by certified trainers following 15 modules. One focus was on 8 protective ‘sources of strength’ and skills for increasing those resources for themselves and other students. Another focus was on engaging ‘trusted adults’ to help distressed and suicidal peers	Developed and used in the same country for two studies and used in different countries without mentioning contextualisation status in another two studies
4.	Youth Aware Mental Health (YAM)	An introduction to mental health is provided by using slides, posters, and a booklet for each participant to keep. The information in the materials contains of six topics used in role‐play and discussions. The topics are: What is mental health, Self‐help advice, Stress and crisis, Depression and suicidal thoughts, Helping a friend in need, and Getting advice	Not mentioned (country of development different from this research)
5.	SESH (South Elgin High School) suicide prevention program	Three days of activities for the students. In the first day, 50‐min presentations by social workers. The information is about Choosing Life: Gail's story, a 14‐min video and a structured discussion that included 13 transparencies presentation. In the second day, students completed 20 quiz questions. On the third day, four trained teachers evaluated the quiz and encouraged discussion about items that students missed	Developed and used in the same country
6.	RAPP (Raising Awareness of Personal Power)	A curriculum‐based suicide education programme that educates students about depression, bipolar disorder, suicidal warning signs, a three‐step process; listen, ask, act of responding and local resources. The programme consists of lectures and activities including interactive games, role‐plays, and the analysis of several stories. Each participant receives a RAPP workbook, a Youth Yellow Pages resource guide, and a yellow Ribbon card	Developed and used in the same country
7.	Psychoeducational prevention program	Two suicide prevention approaches (psychoeducational and peer‐helping) were combined into a single programme. The programme included one meeting of 2 h which developed into two components; one is to increase knowledge and adaptive attitudes, and the other part is to have an impact on coping behaviour. It includes an epidemiological outline of suicidal thoughts, and attempts and how it progresses to suicidality, possible causes, and risk factors of suicide. The peer‐helping programme focused on the identification of warning signs, recognition of signs of suicide, and responding appropriately	Developed and used in the same country
8.	Suicide Awareness Curriculum	Three 40 to 45‐min participatory classes, the first session focused on suicide, attitudes towards suicide and the tunnel thinking that is produced by extreme stress. The second session was about warning signs and seeking help from adults. The third lesson included a video regarding the consequences of failing to respond to a suicidal peer on time. A wallet card with suicide information and local crisis phone numbers was provided to all students	Developed and used in the same country
9.	Teen Mental Health First Aid (tMFHA)	Three sessions of 75‐min classroom presentation, which included PowerPoint presentation, videos, role‐plays, group discussion, small group and workbook activities. The first session focused on an introduction to mental health and problems and impacts on young people, stigma, and types of appropriate help. Session 2 focused on the skills to help a friend in a mental health crisis with a video presenting a teenager experiencing suicidal thoughts followed by a group discussion about helping others while experiencing suicidal thoughts and about confidentiality vs. Safety in suicide. Session 3 was about the importance of early intervention, using tMFHA action plan and resources for help	Developed and used in the same country for preventing different mental health conditions.
10.	Signs of Suicide (SOS) (Aseltine Jr. 2003, Aseltine Jr. and DeMartino 2004, Aseltine Jr. et al. 2007, Schilling et al. 2014, 2016) (*n* = 5)	Youths are taught to recognise of the signs and symptoms of suicide and depression, and to respond to those signs following specific actions called ACT ‘Acknowledge, Care, and Tell’. First, ACKNOWLEDGE the signs of suicide that others display and take them seriously. Next, let the person know that you CARE. Then, TELL this to a responsible adult. Peer leaders promote and model help‐seeking behaviour, and communication with trusted others, and use 8 sources of strength—which include trusted individuals, health promotion activities, and medical care. Peer leaders are trained to develop and conduct whole school messaging activities—posters, videos, presentations, walls of trust, thankfulness challenges, and mental health awareness days. Teaching materials include a video drama depicting the signs of suicide and depression, ways to respond to it and a discussion guide	Developed and used in the same country
11.	Public service announcement	In the billboard condition, participants were asked to imagine that they saw the billboard message while they were driving followed by projecting the message for 5 s. The message contained ‘Prevent Suicide, treat depression‐See your doctor’ and the middle‐aged male was depicted. In the TV ad condition, depression was described as a brain illness, listed salient features of depression and it leads to suicide and urged depressed individuals to seek medical help. The third item was a question that asked the participants what type of information they thought might be useful in preventing suicide	Developed and used in the same country
12.	Counsellor CARE (C‐CARE) and P‐CARE	Based on prevention science theory the interventions are targeted to improve emotion management and coping skills and to increase support seeking and receiving. (1) 1:1 suicide assessment interview (2 h), (2) a brief motivational counselling session to enhance empathy and support, deliver personal information, reinforce positive coping skills and help‐seeking behaviour, and increase access to help, (3) a social network connection intervention to link the youth with a case manager, favourite teacher or both, and to contact a parent or guardian of choice to enhance support, access help and communication among the youth, school personnel and parents (1.5–2 h)	Developed and used in the same country
13.	C‐CARE, CAST	CAST: small group skills training and social support intervention (targeting mood, school performance and drugs). One‐hour session twice a week for 6 weeks (12 h)	Developed and used in the same country
14.	Harry Potter‐based Mental health literacy program	A 3‐month teacher‐delivered intervention included risk factors for emotional distress, protective factors to promote resilience, behavioural intervention for improving mood and promotion of help‐seeking behaviour. Mental health/CBT education included (a) how risk factors contribute to emotional distress and protective factors promote resilience, (b) manifestation of depression and anxiety (c) differences between cognitive distortion and rational thoughts (d) cognitive restructuring techniques (e) behavioural intervention for improving mood and (f) promoting help‐seeking behaviour	Developed and used in the same country
15.	YAM and ProfScreen	YAM: Developed for SEYLE study. Universal intervention with 3 h of role‐play and interactive workshops, a 32‐page booklet to take home, 6 posters displayed in classes, and 2 × 1 h lectures about mental health (total 5 h in 4 weeks). Prof Screen: Developed for SEYLE study. A selective or indicated intervention based on responses to the SEYLE baseline questionnaire. Answers reviewed by HCP, students who screened at or above cut‐offs were invited to mental health clinical assessment and offered referral if necessary	YAM and ProfScreen were developed and used in the same context
16.	PGC	Social support and life skills training for PGC Group I (5 months), Group II (10 months) and Group III (control group). PGC Group I emphasised training the youth to give and receive social support in a closely supervised PGC group setting, PGC group II emphasised transferring social skill support into other school settings and bonding via participation in existing school clubs and activities. Group III was the assessment‐only group	Developed and used in the same country
17.	Suicide prevention curricula	Limited details were provided about the prevention programme. However, it lasted 1.5 h, provided by a teacher (trained) in a classroom, and emphasised support networks, confronting peers and community resources	Not mentioned
18.	Experiential suicide prevention program	It was a guided discussion, focused on important issues of adolescents. The workshop consisted of seven weekly meetings of 2 h duration. Each meeting was focused on one of the following items: (1) depression and happiness, (2) The adolescent and family, (3) feeling of hopelessness, (4) coping with failure, (5) personal thoughts on coping with stress and problem‐solving, (6) coping with suicidal urges and (7) summary and feedback with an optional session of Separation and Loss	Not mentioned
19.	EMPATHY (Silverstone et al. 2015, 2017) (*n* = 2)	1. Guided Internet CBT: High‐risk and moderate‐risk groups received rapid response CBT, Low suicide risk groups were offered a guided Internet CBT programme. The programme was guided by the Resiliency Coaches. Resiliency coaches received a 5‐day training. It was an established internet‐based CBT programme 2. Modified CBT OVK: All students in Grades 7 and 8 received a modified version of a CBT programme designed to reduce depressive symptoms. This programme implemented the first eight sessions (originally 16), a 45‐min classroom lesson for each session	Not mentioned
20.	Oregon Healthy Teens (OHT) Survey	This study conducted the Oregon Healthy Teens Survey to evaluate the growing number of Services through School‐Based Health Centers (SBHCs) already implemented. SBHC is a comprehensive and convenient healthcare service for school children. It is a national effort to provide cost‐effective healthcare to youths. It was conducted in schools having school‐based health centres	Developed and used in same country
21.	SEYLE	It is an interactive workshop. The programme consisted of an opening lecture with three role‐play sessions, and a closing lecture with a discussion—total programme was 5 h. A 25‐page booklet was provided which contained six themes: (1) awareness of mental health, (2) self‐help advice, (3) stress and crisis, (4) depression and suicidal thoughts, (5) helping a troubled friend and (6) getting advice about the contact person	Developed and used in the same countries
22.	HCCI	HCCI was based on a youth leadership model that focused on 4 components of Prevention: Youth empowerment, relationship and team‐building activities, suicide prevention training and community awareness events	Not mentioned
23.	Classroom‐based suicide prevention program	The programme was teaching students the ‘facts’ of suicide emphasising on encouraging the youth to enlist the adults who could assist their suicidal peer. Sources of distress among youth; coping and stress management skills; recognising warning signs and responding to suicide risk; and how to help. Learning activities were short lectures, small group discussions and the presentation of a 20‐min DVD. The film presented young people talking about their past experiences with suicidal behaviour, and demonstration of peer support intervention	Mentioned contextualised from mainstream approach to the conceptualisation, but not clearly explained
24.	LifeAct	It is a school‐based programme for teaching adolescents about signs of depression, depression and other mental illnesses and see themselves as the ‘first line’ of defence in recognising the signs of suicide in themselves or friends. This programme also provides instructions on how to overcome the stigma of mental health problems, help‐seeking, offering help and connecting to the necessary professional help. The programme was delivered using real‐life situation presentations, role‐play, skits and small group activities	Developed and used in the same country
25.	Life skills education	At the community level: Educational interventions were designed and delivered to the youth patrol group on risk factors for suicidal behaviour. A total of 11 sessions, lasting 2 h, were delivered. At the school level: Intervention was concentrated on four specific skills; self‐awareness, empathy, coping with emotions and effective communication. A total of 12 sessions were conducted once a week and the session duration were 50–60 min for each	Not mentioned
26.	LROP	LROP is a cultural enrichment 6‐week programme designed to teach Lumbee Youth about their culture, classes focus on educating the youth on ways to identify factors affecting health and strategies to address these issues in culturally appropriate ways. Without specifically targeting suicide, it targeted improved self‐esteem and social support as a means of suicide prevention	Developed for Lumbee youth and used there only
27.	Mindfulness‐based suicide prevention program	An elective class at a native American School that occurred four sessions per week, each session was 55 min over 10 weeks. Contents were specific mindfulness practice, story, metaphor, experiential activity and discussion	Contextualised the mindfulness of local tribal culture
28.	SafeTALK (Bailey et al. 2017; Kinchin et al. 2019) (*n* = 2)	It is a suicide prevention programme designed for aged 15 years and older to recognise suicide invitations, respond to those invitations and connect the person with further help. Workshops were delivered to about 30 students at a time, facilitated by one SafeTALK trainer in the presence of staff members and counsellors to increase safety. A pocket card containing the suicidal alert steps was provided to the participants at the end of the workshop	Not mentioned
29.	Curriculum‐based suicide prevention program	A total of three programmes were delivered. Programme 1 focused on clinical features of suicidal adolescents and the need to seek professional help, delivered by professionals. Programme 2 emphasised the value of supporting networks in decreasing stress. Programme 3 focused on problem‐solving techniques	Not mentioned
30.	E‐CPR	The 90‐min workshop included components of knowledge and attitude to build suicidal awareness, recognise warning signs, identify barriers, help others using crisis and seek help for themselves	Not mentioned
31.	Erika's Lighthouse and Understanding teen depression	Erika's Lighthouse Foundation created a programme intending to alter adolescents' views on depression and suicide using video presentations, lectures, adolescent discussion panels and larger classroom discussions in school	Developed and used in the same country
32.	Surviving the Teens (King et al. 2011; Strunk et al. 2014) (*n* = 2)	It consists of 50‐min sessions over four consecutive days. Two major risk factors of suicide; Depression and substance use are focused on the programme. It is based on the self‐efficacy model of Bandura's Social cognitive theory. This programme encourages teens to seek help for themselves and their peers who are at risk of suicide	Developed and used in the same country
33.	Campus Connect Training	Training included information regarding college student suicide prevalence, warning signs, strategies for asking students about suicidal ideation and strategies for making referrals. Training was in didactic format, experiential exercises and role‐play	Not mentioned
34.	Yellow ribbon (Freedenthal 2010; Flynn et al. 2016) (*n* = 2)	A 60‐min Student leadership training included a digital slide presentation including the history of the Yellow Ribbon programme, adolescent suicide, prevalence, myths and facts, warning signs and the importance of seeking help. The 50‐min school‐wide assembly included a personal story of Emme's son's suicide and messages about help seeking followed by the distribution of Ask4Help cards	Developed and used in the same country
35.	Sophomore‐level health class	The teacher distributes and reviews written materials on the warning signs of suicide and basic intervention strategies with suicidal peers. Then the following day, the researcher presented a 15‐min video that depicts adolescents who are lonely and need to belong, showing them adolescents' feelings are not unique. The filmstrip was followed by 40‐min structured discussions on healthy and unhealthy messages	Not mentioned
36.	Youth suicide prevention	4 h curriculum on suicide prevention	Not mentioned
37.	SOS education output	A 50‐min lesson including stress awareness, help‐seeking, help‐offering and picture‐book reading. Part one was about introduction to the health centre and content (5 min), and part 2 was about stress and stress coping/SoS output lecture of 25 min. In part 3, students read the picture book called ‘You Are Special’ for 10 min and part 4 was the conclusion (2 min) of the programme	Developed and used in the same country
38.	Reframe IT	15 to 20 min module per week for 8 weeks; an adult host delivering the video diaries made by young people (actors) telling different stories each week. Eight modules followed standard CBT approaches	Developed and used in the same country
39.	LifeSavers	Three days manualised weekend retreats consist of small and large group activities to train the high school students. The contents were teamwork, nonjudgemental listening to other's concerns, recognising peers' suicidal thoughts and enlisting the available help and support	Not mentioned
40.	CBT and peer leadership	Training received by mental health nurses (TKN), Cognitive behaviour therapy (CBT) and Peer Leadership (PL). CBT changes negative thoughts and behaviours into positive ones to reduce negative emotional effects while PL reinforces leadership, community service and social change	Not mentioned
41.	Stop suicide	A 90‐min workshop, containing information about suicide, seeking and offering help, and identification of warning signs and myths of suicide. The workshop was delivered using lecture, discussion, case discussion and quiz methods plus the use of an illustrated book	Developed and used in the same country

Most of the programmes focused on classroom activities to educate the participants about the signs of suicide and recognition of those signs in themselves and others. Despite some similarities, variations were observed in terminology in describing signs of suicide, frequency, and lengths of sessions, trainers (persons who deliver the programme) and programme delivery standards, such as modes of delivery and size of the groups. For instance, one programme had 40–45‐min sessions (Kalafat and Elias 1994), while others ran for 4 h (Wyman et al. 2010; Petrova et al. 2015; Calear et al. 2022; Pickering et al. 2022). Similarly, certified trainers delivered some programmes (Freedenthal 2010; Flynn et al. 2016; Bailey et al. 2017; Kinchin et al. 2019; Hart et al. 2020), while in another study, school teachers delivered a programme after receiving an orientation (Ciffone 1993) (Table 2).

Each programme had a distinct focus. For example, Signs of Suicide (SOS) focused on teaching adolescents to recognise the signs and symptoms of suicide and depression and to respond following the ACT steps ‘Acknowledge, Care, and Tell’ (Aseltine Jr. 2003; Aseltine Jr. and DeMartino 2004; Aseltine Jr. et al. 2007; Schilling et al. 2014, 2016). Source of Strength aimed to train both adolescents and adults in the identification of trusted adults in youth suicide prevention (Wyman et al. 2010, Petrova et al. 2015, Calear et al. 2022, Pickering et al. 2022). Yellow Ribbon based its programme on teaching adolescents about suicide prevalence, myths and facts, warning signs and the importance of seeking help (Freedenthal 2010; Flynn et al. 2016). Surviving the Teens programme focused on depression, suicide and substance use (King et al. 2011; Strunk et al. 2014). While SafeTALK was centred on recognising invitations indicating that a person may be experiencing suicidal thoughts, how to respond to those invitations, and connecting the person with further assistance (Bailey et al. 2017; Kinchin et al. 2019).

### 3.3 Contextualisation of Suicide Prevention Programmes

Almost half (*n* = 20) of the programmes used in various studies were developed and used in the same country (i.e., country of development). Two studies (McGillivray et al. 2021; Calear et al. 2022) utilised the programme developed in another location with similar socioeconomic conditions; however, these studies did not report on the contextualisation process (Calear et al. 2022). Another study (Calear et al. 2021) discussed contextualising the Source of Strength programme for use among males, and two studies (White and Morris 2010; Le and Gobert 2015) reported programmes that were customised. However, few details were recorded about the steps involved in the contextualisation. One study reported a participatory method of developing a programme related to the context (Langdon et al. 2016). Many studies (Silverstone et al. 2015, 2017; Bailey et al. 2017; Kinchin et al. 2019; Nasution et al. 2019; Wise 2023) did not mention programme development (Table 2).

### 3.4 Effects of the Programmes

Suicidal behaviours (suicide plan, ideation, attempt and risk) were the most frequent outcomes measured across the studies (Table 3). A variety of tools were used to measure the outcomes (suicidal behaviour, help seeking, knowledge, attitude and coping). Some of the repeatedly used tools were Youth Risk Behaviour Survey (YRBS) (Aseltine Jr. and DeMartino 2004; Aseltine Jr. et al. 2007; Schilling et al. 2014, 2016), General Help‐Seeking Questionnaire (GHSQ) (Calear et al. 2021, 2022; McGillivray et al. 2021), Actual Help‐Seeking Questionnaire (AHSQ) (Calear et al. 2021, 2022, McGillivray et al. 2021), High School Questionnaire (Thompson et al. 2000, 2001; Eggert et al. 2002; Hooven et al. 2010, 2012), Patient Health Questionnaire (PHQ) (Le and Gobert 2015; Silverstone et al. 2015, 2017), Hospital Anxiety and Depression Scale (HADS) (Silverstone et al. 2015, 2017) and Beck Hopelessness Scale (BHS) (Portzky and van Heeringen 2006; Robinson et al. 2016). Studies also reported using self‐constructed tools relevant to the prevention programme they used (Ciffone 1993; Kalafat and Elias 1994; Cigularov et al. 2008).

**TABLE 3:** Effects of programmes on suicidal behaviour, help‐seeking and other outcomes.

Author, Year	Outcomes	Effects
Bockhoff et al. (2023)	Help seeking	No significant effect, *p* = 0.286
	Help giving	Increased *p* = 0.022
Calear et al. (2021)	Help‐seeking intention	Increased help‐seeking from friends *t* = 1.217 (pretest) and 2.437 (posttest) *p* = 0.015
	Confidence to support and help‐seeking stigma	No significant effects (*p* = 0.921 and 0.158)
Wyman et al. (2010)	Perceptions of adult help for suicidal peers	Significant positive perceptions (ES = 0.63; 95% CI = 0.29, 0.97), and on norms for help‐seeking from adults (ES = 0.58; 95% CI = 0.24, 0.91)
	Coping measures	No significant effect *p* = 0.966
Calear et al. (2022)	Suicidal ideation	No significant effects, *p* = 0.942
	Help seeking	No significant effects, *p* = 0.261
Pickering et al. (2022)	Suicidal ideation, attempt	FNOL had lower proportion of SI (*M* = −0.15, SE = 0.03), POL and FNOL‐CI Had lower attempt (*M* = −0.10 and −0.14 respectively)
	Direct communication	POL increased significantly, *p* = < 0.001
Petrova et al. (2015)	Help seeking	Enhanced help‐seeking acceptability in students with Suicidal Ideation Est (SE) 0.342 (0.103) *p* = < 0.001), enhanced attitudes about adult help‐seeking Est (SE) 0.224 (0.082) *p* = < 0.01 and could identify specific trusted adult *p* = < 0.05
	Coping	Improved coping EST (SE) 0.238 (0.083), *p* = < 0.01
McGillivray et al. (2021)	Suicidal ideation and attempt	Significantly decreased SI 51.6% at baseline to 39.9% 6 months follow‐up *F* (2, 329.6) = 8.45, *p* = < 0.001 and attempt decreased from 8.7% in baseline, 7.6% in 3 months and 7.8% in 6 months
	Help‐seeking	Significant changes in help seeking *M* = 38.07, SD 11.12 in baseline to *M* = 39.95, SD = 10.24 and *M* = 40.56, SD = 11.21 in 6 months *p* = < 0.001
Ciffone (2007)	Attitude towards suicide	Significantly high posttest scores among treatment groups with mean difference of 1.41 in School 1 and 1.42 in School 2 *p* = < 0.001
Cigularov et al. (2008)	Knowledge	Significantly improved, *t* = 12.97, *p* = < 0.01
	Attitude	Significantly improved, *t* = 4.28, *p* = < 0.01
	Self‐efficacy	Significantly improved, *t* = 9.28, *p* = < 0.01
Portzky and van Heeringen (2006)	Knowledge	Significant positive effects on comparing pre‐ and posttest scores with *F* (1,164) = 44.771; *p* = < 0.001. The experimental group had higher scores *M* = 20.83, SD = 2.58 than the control group *M* = 18.25, SD = 2.385
	Attitude	No significant effect, *p* = 0.653
	Coping	No significant effect, *p* = 0.869
Kalafat and Elias (1994)	Knowledge	Significant improvement (*F* [7,234] = 12.82, *p* < 0.001)
	Help and talk attitude	Significant improvement (*F* [14,225] = 1.87, *p* = < 0.03)
Hart et al. (2020)	Recognition of suicidality	Increased in tMFHA group from 11% baseline to 16% posttraining and 13% in 12 months, whereas dropped in PFA group OR = 1.97 95% CI (1.14, 3.39)
	Adequate suicide first aid	Increased in tMFHA group from 14% in baseline to 62% in posttraining and 31% in 1‐year follow‐up whereas decreased in PFA group OR = 35.40 95% CI (19.86,63.14)
	Avoid asking about suicide	Significantly reduced in tMFHA group as compared to PFA OR = 0.13 95% CI (0.30, 0.721)
Aseltine Jr. et al. (2007)	Reported Suicidal attempt	Treatment group were about 40% less likely to report suicidal attempts as compared to the control group *B* = −0.47, Se 0.16 *p* = < 0.05
	Knowledge	Increased *B* = 0.59, SE = 0.05 *p* = < 0.05.
	Attitude	More favourable attitude towards suicide *B* = 0.16 SE = 0.03 *p* = < 0.05
	Help‐seeking	No significant effect
Aseltine Jr. and DeMartino (2004)	Suicide attempts	Reduced by 40% *B* = −0.467, *p* = < 0.05
	Knowledge	Increased *B* = 0.689, *p* = < 0.05
	Attitudes	More favourable attitude *B* = 0.255 *p* = < 0.05 as compared to control group
	Help seeking	No significant effect, *p* = 0.138
Aseltine Jr. (2003)	Help seeking	Increased in number after the programme *M* = 6.79 SD = 0.87 in the past year vs. *M* = 10.63 SD = 1.43 in the past 30 days. The mean number of visits of students to seek help on behalf of friends also increased from *M* = 3.26 to *M* = 3.78
Schilling et al. (2014)	Knowledge	Significantly increased with effect size of 0.40, *p* = < 0.05
	Attitude	No significant effect
	Help‐seeking	No significant effect
Schilling et al. (2016)	Suicide attempt	Intervention group 64% less likely to report a suicide attempt (OR = e‐1.02 = 0.36)
	Attitude	Significantly improved *p* = < 0.05
	Suicide plan	Reduced by 75%
Klimes‐Dougan et al. (2009)	Knowledge	Increased in knowledge among TV viewers *p* = < 0.01
	Help‐seeking	No significant effects, *p* = ≤ 0.1
Hooven et al. (2010)	Suicidal behaviours	No significant different effects
	Personal efficacy	No significant effects of the programme
Hooven et al. (2012)	Suicidal behaviours	A combination of C and P‐CARE was effective in decreasing suicidal ideation *B* = −1.451 *p* = < 0.001, suicidal threats *B* = −0.556 *p* = < 0.05
	Personal efficacy	A combination of C and P‐CARE was effective in increasing problem‐solving coping *B* = 0.570 *p* = < 0.01, personal control *B* = 0.687 *p* = < 0.001
Thompson et al. (2001)	Suicidal behaviours	CAST and C‐CARE both were effective in decreasing suicidal ideation, (*β* = 0.341 CAST, 0.329 C‐CARE *p* = < 0.05) 10 weeks and (*β* = 0.032 CAST 0.028 C‐CARE *p* = < 0.05) in 9‐month follow‐up
	Attitude towards suicide	CAST was effective in improving attitudes towards suicide (*β* = −0.292, *p* = < 0.05 in 10 weeks and *β* = 0.30 *p* = < 0.05 in 9‐month follow‐up)
	Coping	CAST and C‐CARE both were effective in enhancing problem‐solving coping (*p* = < 0.01 in 10 weeks and < 0.05 in 9‐month follow‐up) and sustaining personal control (*p* = < 0.05)
Eggert et al. (2002)	Suicide risk	No significant effect
	Personal efficacy	No significant effect
Klim‐Conforti et al. (2021)	Suicidality	A significant reduction in suicidality *t* = −2.60, *p* = 0.01
	Life problems	Life Problems Inventory (LPI) significantly decreased among the intervention group (*t* = 5.28, *p* = < 0.001)
Wasserman et al. (2015)	Suicide attempts	YAM was significantly associated with decreasing suicide attempts at 12‐month follow‐up (OR 0.45, *p* = 0.014) compared with control
Thompson et al. (2000)	Suicide Risk	There was a significant negative effect of peer group support (*B* = −0.38, *t* = 3.40, *p* < 0.001) on suicide risk behaviours for Group II
	Peer support	Peer support was related to personal control (*B* = 0.39, *t* = 2.07, *p* = < 0.05) for Group I only. Teacher support had a significant positive, effect on peer support (*B* = 56, *t* = 3.35, *p* = < 0.001, and *B* = 0.71, *t* = 5.58, *p* < 0.001, for Groups I and II, respectively)
Vieland et al. (1991)	Suicide attempts	No significant effect
Orbach and Bar‐Joseph (1993)	Suicidal tendency	Significantly decreased suicidal tendency *F* = 7.08, *p* = < 0.05
	Ego identity	Improved ego identity *F* (1,17) = 4.80 *p* < 0.05
	Coping	Improved coping *F* (1,17) = 6.75, *p* = < 0.05
Silverstone et al. (2015)	Suicidality	In CBT group Suicidality was decreased by 8% with *p* = < 0.001 in all groups. In modified OVK group, no significant effect was observed.
Silverstone et al. (2017)	Suicidality	Significantly decreased Suicidal risk, *p* = < 0.001, High and medium risk was decreased to 3.2% in 3 months, 3.3% in 7 months and 2.8% in 15‐month follow‐up. Similarly, any suicidal risk was decreased from 7.3% to 5.9% in 3 months and 7 months and to 5% in 15‐month follow‐up, *p* = < 0.001
Paschall and Bersamin (2018)	Suicidal behaviour	Increased availability of mental health services was associated with a reduction in suicidal ideation (26%) (OR 0.84, *p* = < 0.01) and suicide attempts (32%) (OR 0.82, *p* = < 0.001)
Wasserman et al. (2012)	Suicidal behaviour	Development of networks with the clinical sectors, that facilitated the treatment of students in distress; instructors and parents reported changes in the mood and behaviours of the students
Antonio et al. (2020)	Suicide prevention resources	Five programme impacts were identified based on themes, aligned with an adapted socioecological model (SEM). Youth leaders gained skills and incorporated those skills into their youth leader identity, youth leaders were recognised as ‘eyes and ears’ of their peers and community, and they were also considered as resources and safety net for suicide prevention in the community
White and Morris (2010)	Perceptions about suicide	Common concepts on youth suicide were considered as unimaginably sad, tragic, regrettable, personal event and most often linked to depression and/or crisis. Participants expressed prevention programmes should be discussion‐based, it cannot be conceptualised through universal terms, individual and cultural variation exist
Orlins (2023)	Knowledge	Significantly improved in 2 weeks and 10 weeks; mean scores were 7.18 vs. 6.20 in 2 weeks and 7.08 vs. 6.20 in 10‐week posttest *p* = < 0.05 for both
	Help‐seeking	Significantly improved in 10‐week follow‐up, *X* = 3.134, *p* = 0.077 in 2 weeks and *X* = 4.849, *p* = 0.028 in 10 weeks
Orlins (2023)	Suicide and help‐seeking	Four identified super‐ordinate themes were, (1) desire to engage in discussion on mental health and suicide: Participants believed that it is hard to share but beneficial to engage in discussion. (2) Favourable and unfavourable help‐seeking strategies: Participants identified exercise and journaling as self‐help strategies, many participants expressed peers as the first choice of help‐seeking resources, and help‐seeking from adults was expressed as a barrier. A few participants also expressed fear of getting help from hotline numbers. Some participants also expressed awkwardness in seeking help from friends. (3) deciding whether suicide risk is real: Participants expressed much of their friends' behaviour as attention seeking. Suicidal risk is muddied by commonly expressed words such as ‘killing myself’ or ‘kill yourself’, and such words create confusion. Some participants expressed, coping as humour can hurt the person who is suffering. Some participants expressed confusing criteria of depression that requires 2 weeks to diagnose while someone feeling sad now, and (4) role of stressors in mental illness
Langdon et al. (2016)	Suicidal behaviours	Youth recognised the importance of parental role in mental health and enculturation, the programme was well received, and monthly cultural classes demonstrated a high level of connectedness, 90.1% of the participants wanted to continue participating in the programme. Regular participation showed decreased suicidal ideation and increased protective factors. However, the change was not statistically significant
Le and Gobert (2015)	Suicidal behaviour	Lowered impulsivity, improvement in self‐regulation measures and suicidal intention were decreased by 66%, all the participants did not have suicidal ideation or wish to die during the posttest as compared to 44% who had the thoughts in the pretest. 81% responded positive response on making friends for connectedness. In one of the themes, many youths reported choosing to share personal histories was very important to establish trust
Bailey et al. (2017)	Suicidal behaviour	Decreased significantly with odds of 0.30 95% CI = 0.10–0.91 in 4 weeks
	Knowledge	Significantly increased *p* = < 0.0001 and maintained until 4 weeks
	Help‐seeking, and confident and willing to help	Significantly improved immediately after the programme *p* = < 0.0001 and 4 weeks *p* = < 0.05. Confidence and willingness to help were increased immediately but were not sustained
Kinchin et al. (2019)	Knowledge	Increased from baseline to immediately after the programme (*t* [21] = 8.18, *p* = < 0.001) but, did not maintain 4 weeks later
	Confidence to help	Increased from baseline to immediate follow‐up (*t* [21] = 5.25, *p* = < 0.001) and maintained the almost same level in 4‐week follow‐up
	Willingness to help and help‐seeking	Significantly increased following the programme (21) = 3.03, *p* = 0.006) and slightly increased in 4‐week follow‐up. The likelihood of help‐seeking was significant on completion and after 4 weeks of the programme (*t* [21] =2.18, *p* = 0.044, and t [9] = 2.83, *p* = 0.026)
	Most common words for depression and barriers to help‐seeking	Students recognised some of the most used words for depression were ‘spending more time by themselves’, ‘don't go out’ self‐harm or suicide experience; distancing from others; sadness; isolation; anxiety; and negativity, as ‘negative talking’, ‘negative attitude or approach to life’. Embarrassment was recognised as the main barrier of help seeking and the least important barrier was physical distance. The barrier ‘concern about what treatment could involve’ was changed over time *p* = 0.032
Shaffer et al. (1991)	Knowledge and help‐seeking attitudes	No significant change was observed
Wise (2023)	Confidence in accessing help	Significant improvement with Moderate effect size *r* = 0.56
	Crisis intervention skills	Significant improvement with moderate effect size *r* = 0.50
White (2015)	Suicidal thoughts	A significant change was observed in reporting depression and suicidal thoughts in a friend *M* pretest = 4.64, *M* posttest = 4.72, *t* (330) = −2.24, *p* = < 0.05
	Help seeking	Sense of efficacy in helping peers did not have significant change. Help seeking was reported significantly high during the posttest pretest *M* = 3.12, posttest *M* = 3.57, *t* (330) = −7.47, *p* = < 0.001
	Knowledge and understanding	Significantly Improved, mean pretest 3.89 vs. posttest 4.17, *t* (330) = −14.10, *p* = < 0.001. Significant positive changes in the understanding of suicide, *p* = < 0.001
Strunk et al. (2014)	Knowledge	Knowledge of suicide significantly improved *F* (1,1624) = 9.236, *p* = 0.002. Knowledge of warning signs [*F* (1,1624) = 9.660, *p* = 0.002], and knowledge of myths and facts [*F* (1,1624) = 23.264, *p* = < 0.001]
	Confidence in helping	Improved confidence in helping suicidal friend *F* (1,1502) = 121.409, *p* = < 0.001
Pasco et al. (2012)	Suicidal ideation	Suicidal Ideation Reporting Index score was decreased from pre‐ to posttraining from *M* = 77.71 to 68.64 [*F* (1, 21) = 24.00, *p* = < 0.01]
	Knowledge	Significantly improved, *p* = < 0.05
	Crisis response skill	Improved, *p* = < 0.01 but, significance was lost after adding experiential activities
	Connect during crisis	Increased ability to connect to students in crisis *p* = < 0.05
Freedenthal (2010)	Suicidal ideation	Not improved
	Help seeking	Calling a crisis hotline increased significantly from 2.2% to 6.9% *z* (135) = −2.0, *p* = < 0.05), other 11 types of help‐seeking behaviours remained unchanged
Ciffone (1993)	Confidence in counselling	Significantly improved *p* = < 0.034
	Not keeping peers' suicidal thoughts secret	Significantly improved *p* = < 0.004
	Connecting peers to help	Significantly improved *p* = < 0.001
	Telling suicidal ideation to friends	Significantly improved *p* = < 0.006
	Suicide is the solution	No effect
Nelson (1987)	Knowledge	Significant improvement pretest *M* = 32.20, posttest *M* = 35.48 *p* = < 0.001
	Attitude	Significant improvement pretest *M* = 33.86, posttest *M* = 34.95 *p* = < 0.01
Ogawa et al. (2022)	Self‐worth	No effect
	Worries	Significantly decreased *Q* = 9.75, *p* = 0.008
	Reliable adults	Adults whom you can talk to at any time significantly changed to *p* = 0.048
Robinson et al. (2016)	Suicidal ideation	Significantly decreased *t* (20) = 6.2, *p* = < 0.0005
King et al. (2011)	Suicidal behaviours	Significantly decreased suicidal ideation *p* = 0.035, plan *p* = 0.003, attempt 0.011
	Identification of trusted adults	Identification of trusted adults to share thoughts significantly increased *p* = < 0.001
	Telling suicidal thoughts to other	Increased telling adults own suicidal thoughts *p* = < 0.001 and peers' suicidal thoughts *p* = 0.022
Walker et al. (2009)	Knowledge and attitude	Significantly improved pretest *M* = 97.44, posttest *M* = 103.72 *d* = 0.85, *p* = < 0.001
Flynn et al. (2016)	Knowledge	Increased by 20.9%, pretest correct response 65.9% to posttest 86.8% *z* = 37.7, *p* = < 0.001
	Helping others	Improved significantly, *v* = 0.339, *p* = < 0.001
	Help‐seeking	Improved significantly *v* = 0.243 to *v* = 0.356, *p* = < 0.001
Nasution et al. (2019)	Suicidal ideation	Significantly decreased In Group I which received a combination of three programmes it declined from 4.79 to 0.21 (*p* < 0.05), while TKN only group had decreased from 3.98 to 2.65 *p* = < 0.001 for both groups
Baggio et al. (2022)	Coping	No significant effects
	Help‐seeking	Significant effects in help seeking (*b* = −0.27, 95% CI −0.43, −0.11, *p* = 0.001) confidence in help seeking (*b* = −0.36 95% CI −0.50, −0.21, *p* = < 0.001), intention to seek help (*b* = −0.29, 95% CI −0.50 to −0.07, *p* = 0.010) and knowledge of help‐seeking resources (*b* = −0.39, 95% CI −0.58, −0.30, *p* = < 0.001)

Of the total 28 studies that measured the effectiveness of suicide prevention programmes on suicidal behaviours, 23 (82.1%) reported significant positive effects. Knowledge of suicide was assessed in 16 studies, and 15 (93.8%) reported significant improvement. Help‐seeking was measured in 18 studies, and 12 (66.7%) reported on the effectiveness of the programmes in improving help‐seeking intentions or behaviours. Similarly, the impact of the suicide prevention programme on attitude was measured by 11 studies, and 9 (81.8%) reported effectiveness. The impact of the intervention on coping with suicidal thoughts was evaluated in six studies, and three (50%) studies observed effectiveness (Orbach and Bar‐Joseph 1993; Thompson et al. 2000; Petrova et al. 2015). The study with the largest sample size (*n* = 11 110), across 168 schools in 10 European countries, reported that implementing YAM‐reduced incidents of suicide attempts (odds ratios [OR] 0.45, *p* = 0.014) and severe suicidal ideation ([OR] 0.50, *p* = 0.025), compared to ProfScreen at 12‐month follow‐up. It showed promising effects in reducing suicide; however, implementation involves logistical complexities and financial costs requiring a separate YAM centre to be established for implementation (Wasserman et al. 2015). Furthermore, self‐efficacy, offering help to others, confidence and willingness to help were reported to be positively impacted by suicide prevention programmes across the studies (Hooven et al. 2012; Kinchin et al. 2019; Hart et al. 2020; Ogawa et al. 2022; Wise 2023). None of the studies in this review reported adverse effects of the programmes.

The most frequently used programme, SOS, was reported to be consistently effective in reducing suicidal behaviour across the studies. Nevertheless, this programme was inconsistent in improving attitudes and help‐seeking behaviours (Aseltine Jr. 2003; Aseltine Jr. and DeMartino 2004; Aseltine Jr. et al. 2007; Schilling et al. 2014, 2016). The second most frequent programme, Source of Strength, also reported inconsistent findings in reducing suicidal behaviours but was reported to be effective in improving help‐seeking (Wyman et al. 2010; Petrova et al. 2015; Calear et al. 2022; Pickering et al. 2022). The Yellow Ribbon programme was used by two studies and reported effectiveness in improving help‐seeking behaviour (Freedenthal 2010; Flynn et al. 2016). However, one of the two studies found that the Yellow Ribbon programme had no significant effect on suicidal ideation (Freedenthal 2010). Two studies used SafeTALK programmes and reported effectiveness in increasing knowledge, help seeking and confidence to help across both studies (Bailey et al. 2017; Kinchin et al. 2019) and effectiveness in reducing suicidal behaviour (Bailey et al. 2017). The Surviving Teen reported effectiveness in improving knowledge and help seeking for suicidal behaviours across two studies (King et al. 2011; Strunk et al. 2014). In many programmes reported as being effective, one outcome variable improved but did not affect another outcome of interest.

Two of the qualitative studies included in this review reported the use of the word suicide and its description in different circumstances as barriers to help seeking or offering help during suicidal thoughts. For example, casual use of words like ‘mental problems’ and ‘killing oneself’ may lead to confusion for people who want to help people experiencing suicidal thoughts (Langdon et al. 2016; Orlins 2023). Further one of the qualitative studies that aligned with an adapted socioecological model reported after receiving the training, youth leaders were recognised as the ‘eyes and ears’ of their peers and acted as a safety net for suicide prevention among youth (Langdon et al. 2016). In addition, in the qualitative case study design, participants stated the existence of individual and cultural variations in understanding suicide and that it cannot be conceptualised using universal terms (White and Morris 2010).

## 4 Discussion

A systematic review was conducted that included all types of study designs to investigate suicide prevention programmes and their effectiveness with adolescents in nonclinical settings. In our review, the sample size included individual studies with participant samples as low as eight, which are inconsistent with previous reviews (Robinson et al. 2013, 2018; Liljedahl et al. 2023). Studies with low sample sizes were included because this review was not limited to Experimental and Quasi‐experimental research designs in contrast to previous reviews (Kalafat and Ryerson 1999; Miller et al. 2009; Robinson et al. 2013, 2018; Liljedahl et al. 2023).

The review aimed to identify the available suicide prevention programmes for adolescents globally. However, most of the identified programmes were from Organisation for Economic Cooperation and Development (OECD) countries. There were very few from upper‐middle‐income countries (Kalafat 2003; Robinson et al. 2018; Liljedahl et al. 2023) and no studies from low–middle‐income countries (LMICs). This finding was consistent with previous similar systematic reviews (Robinson et al. 2018; Pearce et al. 2022; Liljedahl et al. 2023). However, one systematic review that included studies conducted in healthcare settings found one study from Sri Lanka (Robinson et al. 2018). The possible explanation for observing the dearth of literature from LMICs may be a reflection of political and social barriers around mental health and suicide (Pearce et al. 2022). Some of the reported barriers from LMICs are lack of political prioritisation and lack of appropriate support from family and friends for mental health conditions (Renaud et al. 2022).

Previous research focused on cultural, social and racial differences in suicidal behaviour and help‐seeking among young people suggests the need to adjust the programme (Goldston et al. 2008). Nevertheless, there was one qualitative study based on multilayer collaboration for suicide prevention in our review that reported the importance of customisation of the programme by involving multiple layers of stakeholders for a sustainable suicide prevention programme. Stakeholders included youth, community organisations and healthcare workers (Langdon et al. 2016). Previous research demonstrated a gap in considering the dissemination and implementation of school‐based suicide prevention programmes that were exported for use in international communities or populations of another sociocultural background (Goldston et al. 2008; Liljedahl et al. 2023). However, the programmes transferred to the international community did not mention any cultural or social adjustment of the programme in our review (McGillivray et al. 2021; Calear et al. 2022). Yet, a few studies did report adjustment of programmes for a particular gender or a small community within the same country (White and Morris 2010; Langdon et al. 2016; Calear et al. 2021). Considering the value of culturally tailored programmes, the scantiness of literature and the recommendation of a previous study (Renaud et al. 2022), it is imperative to increase research in this area.

Even though many studies reported the effectiveness of programmes on suicidal behaviour, help‐seeking, knowledge, attitude and coping with thoughts of suicide, no studies included in this review measured all these outcomes together. Follow‐up periods across the studies ranged from immediately after the intervention to 18 months after, and it is inconclusive as to whether the longer‐term effects were sustained past this point. However, the sustainability of programme effects may be enhanced by training local community members to be suicide prevention advocates.

The heterogeneity of the studies and diversity of the programs, assessment tools, standards of programme delivery and follow‐up times across the studies made it difficult to demonstrate that any suicide prevention programme was the gold standard for its effectiveness in reducing suicidal behaviour and improving knowledge, attitudes, as well as promoting help‐seeking and coping skills when experiencing suicidal thoughts. It highlights the importance of generating more promising evidence and developing guidelines to prevent adolescent suicide.

Suicide is a public health concern and requires collaborative efforts from many levels of society to develop sustainable adolescent suicide prevention programmes (Pearce et al. 2022). A multisectoral approach is essential to minimise the burden of suicide (Purohit and Narayanan 2022). Contemporary programmes are focused on testing effectiveness based on classroom‐related activities. In our review, only one programme (Langdon et al. 2016) focused on a multilayer collaborative approach to develop a suicide prevention programme. A previous systematic review also found a limited number of studies related to multisectoral collaboration in suicide prevention research (Pearce et al. 2022).

This review sheds light on the need for evidence from LMICs. Similarly, it highlighted the need to replicate the studies to generate culturally appropriate and sustainable high‐level evidence. Despite having important strengths, being a comprehensive review, there are a few limitations. One limitation is that we excluded studies from healthcare facilities, which might have impacted access to more articles from LMICs. Another limitation may be that excluding studies published in languages other than English might result in missing valuable information. Including various types of designs and not using standardised tools for outcome assessment by all studies in the review restricted us from statistical integration of the findings.

## 5 Conclusion

This review identified many studies evaluating many different suicide prevention interventions for adolescents worldwide. Most of the intervention programmes were developed and used in the same nation. Very few programmes reported customisation of the programme in different contexts and did not report the details of the process. Most of the studies reported the effectiveness of the programme they used. However, none of the programmes measured effectiveness in reducing suicidal behaviours and improving help‐seeking, knowledge, attitude and coping in one single study.

## 6 Relevance to Clinical Practice

Observing the scantiness of evidence from LMICs demonstrates a need to generate evidence from these settings and a need for customisation of suicide prevention programmes for use in culturally and socially diverse contexts. In addition, methodological replication using the same prevention programme is required to confirm the promising effects of any of the programmes. Mental health nurses working in diverse, resource‐constrained countries must be cognisant of these gaps and advocate for the development and implementation of culturally relevant suicide prevention programmes.

## Author Contributions

**Rita Pokharel Poudel:** conceptualisation, methodology, investigation, data curation, validation, formal analysis, visualisation, writing – original draft. **Sheeja Perumbil Pathrose, Diana Jefferies:** conceptualisation, methodology, data curation, validation, writing – review and editing.**: Lucie M. Ramjan:** conceptualisation, methodology, data curation, validation, formal analysis, writing – review and editing, supervision.

## Conflicts of Interest

The authors declare no conflicts of interest.

## Supporting information


Appendix S1.

Appendix S2.

Appendix S3.

Appendix S4.

Data S1.

## Data Availability

The data that support the findings of this study are available from the corresponding author upon reasonable request.
