# Supplementary material for: Effectiveness of Suicide Prevention Programmes Among Adolescents and Sociocultural Adaptation of Programmes: A Systematic Review
Source: Int J Ment Health Nurs. 2025 Apr 10;34(2):e70038. doi: 10.1111/inm.70038 (PMC11984072; doi:10.1111/inm.70038)
Supplement: Supplementary file 3 — Appendix S3. [file INM-34-0-s001.docx]

**Appendix 3- Excluded articles and reasons for exclusion**

| **Exclusion code** | **Title** | **Authors** | **Published Year** | **Journal** |
| --- | --- | --- | --- | --- |
| Outcomes | Self-help tool: Risk management and improving mental wellbeing of New Zealand adolescents | Frederickson D; Shepherd M; Te Maro B; Hetrick S. | 2021 | Advances in Mental Health |
| Outcomes | An exploration around peer support for secondary pupils in Scotland with experience of self-harm | Shepherd V. | 2020 | Educational Psychology in Practice |
| Outcomes | The Signs of Suicide (SOS) Prevention Program pilot study: High school implementation recommendations | Volungis AM. | 2020 | North American Journal of Psychology |
| Outcomes | Suicidal behaviour in adolescents: Educational interventions in Mexico | Arenas-Monreal L; Hidalgo-Solórzano E; Chong-Escudero X; Durán-De LC; Nancy L. González-Cruz; Pérez-Matus S; Valdez-Santiago R. | 2022 | Health & Social Care in the Community |
| Outcomes | Examining the factors that impact utilization of school-based mental health services for Mexican American adolescents from a rural, borderland community during the time of COVID-19: A quantitative study | Cervantes MA. | 2022 | Thesis |
| Population | Managing youth suicide risk at school: Prevention practices, challenges, and future implications | Fortier AM. | 2021 | Thesis |
| Outcomes | Engaging school mental health professionals to deliver evidence-based interventions to Hispanic families | Tapia MI; Ocasio MA; Estrada Y; Pantin H; Prado G. | 2017 | Health Promotion Practice |
| Outcomes | Service use by at-risk youths after school-based suicide screening | Gould MS; Marrocco FA; Hoagwood, K; Kleinman M; Amakawa L; Altschuler E. | 2009 | Journal of the American Academy of Child & Adolescent Psychiatry |
| Population | The sustained reduction of youth suicidal behavior in an urban, multicultural school district | Zenere FJ; Lazarus PJ. | 2009 | School Psychology Review |
| Population | A peer counselor crisis intervention training program to help prevent adolescent suicide | Martin D; Martin M; Barret C. | 1987 | Techniques |
| Outcomes | Short-term prediction of suicidal thoughts and behaviors in adolescents: Can recent developments in technology and computational science provide a breakthrough? | Allen, Nicholas B; Nelson, Benjamin W; Brent, David; Auerbach, Randy P. | 2019 | Journal of Affective Disorders |
| Population | Suicide Concern Reporting among Utah Youths Served by a School-Based Peer-to-Peer Prevention Program | Wright-Berryman J; Hudnall G; Bledsoe C; Lloyd M. | 2019 | Children & Schools |
| Population | A Community-Based Response to a Suicide Cluster | Lai, Carmen C.S; Law, Yik Wa; Shum, Angie K. Y; Ip, Flora W.L; Yip, Paul S.F. | 2019 | Crisis: The Journal of Crisis Intervention & Suicide Prevention |
| Population | Exposure to a mnemonic interferes with recall of suicide warning signs in a community-based suicide prevention program | Bryan, C.J; Steiner-Pappalardo, N; Rudd, M. D. | 2009 | Suicide & Life-Threatening Behavior |
| Population | Suicide prevention through online gatekeeping using search advertising techniques: a feasibility study | Sueki H; Ito J. | 2015 | Crisis: The Journal of Crisis Intervention & Suicide Prevention |
| Population | Assessing intervention effects in a community-based trial to reduce self-harm: a methodological case study | Congdon, P; Clarke, T. | 2005 | Public Health |
| Population | Hope and the interpersonal-psychological theory of suicidal behavior: replication and extension of prior findings | Anestis, Michael D; Moberg, Fallon B; Arnau, Randolph C. | 2014 | Suicide & Life-Threatening Behavior |
| Outcomes | A support group intervention for at-risk female high school students | Houck, G. M; Darnell, S; Lussman, S. | 2002 | Journal of School Nursing |
| Population | Suicide prevention in schools: what can and should be done | Potter, L; Stone, D.M. | 2003 | American Journal of Health Education |
| Outcomes | teen Mental Health First Aid: 12-month outcomes from a cluster crossover randomized controlled trial evaluation of a universal program to help adolescents better support peers with a mental health problem | Hart, L.M; Morgan, A.J; Rossetto, A; Kelly, C. M; Gregg, K; Gross, M; Johnson, C; Jorm, A.F. | 2022 | BMC Public Health |
| Population | Analysing the Impacts of Financial Support for Regional Suicide Prevention Programmes on Suicide Mortality Caused by Major Suicide Motives in Japan Using Statistical Government Data | Nakano, T; Hasegawa, T; Okada, M. | 2021 | International Journal of Environmental Research & Public Health |
| Outcomes | Youth Voice in Suicide Prevention in Hawai'i | Trinh, T; Goebert, D. | 2020 | Hawaii Journal of Health and Social Welfare |
| Population | Kognito's Avatar-Based Suicide Prevention Training for College Students: Results of a Randomized Controlled Trial and a Naturalistic Evaluation | Coleman, D; Black, N. Ng, J; Blumenthal, E. | 2019 | Suicide & Life-Threatening Behavior |
| Outcomes | Analysis of adolescents' opinions on suicide prevention | Kielan, A; Cieslak, I. Skonieczna, J; Olejniczak, D; Jablkowska-Gorecka, K; Panczyk, M; Gotlib, J; Walewska-Zielecka, B. | 2019 | Psychiatria Polska |
| Population | Employing a sequential multiple assignment randomized trial (SMART) to evaluate the impact of brief risk and protective factor prevention interventions for American Indian Youth Suicide | O'Keefe, V.M; Haroz, E.E; Goklish, N; Ivanich, J; Cwik, M.F; Barlow, A. | 2019 | BMC Public Health |
| Population | Evaluation of a Peer-Led Implementation of a Suicide Prevention Gatekeeper Training Program for College Students | Samuolis, J; Harrison, A.J; Flanagan, K. | 2020 | Crisis: Journal of Crisis Intervention & Suicide |
| Setting | LET's CONNECT community mentorship program for youths with peer social problems: Preliminary findings from a randomized effectiveness trial | King, C.A; Gipson, P.Y; Arango, A; Foster, C.E; Clark, M; Ghaziuddin, N; Stone, D. | 2017 | Journal of Community Psychology |
| Population | Identification, Response, and Referral of Suicidal Youth Following Applied Suicide Intervention Skills Training | Ewell Foster, C.J; Burnside, A.N; Smith, P.K; Kramer, A.C; Wills, A; King C.A. | 2016 | Suicide & Life-Threatening Behavior |
| Population | A controlled trial of implementing a complex mental health intervention for carers of vulnerable young people living in out-of-home care: the ripple project | Herrman, H; Humphreys, C; Halperin, S; Monson, K; Harvey, C; Mihalopoulos, C; Cotton, S; Mitchell, P; Glynn, T; Magnus, A; Murray, L; Szwarc, J; Davis, E; Havighurst, S; McGorry, P; Tyano, S; Kaplan, I; Rice, S; Moeller-Saxone, K. | 2016 | BMC Psychiatry |
| Population | Student assistance program outcomes for students at risk for suicide | Biddle, V.S; Kern, J 3rd; Brent, D.A; Thurkettle, M.A; Puskar, K.R; Sekula, L.K. | 2014 | Journal of School Nursing |
| Population | Science from evaluation: testing hypotheses about differential effects of three youth-focused suicide prevention trainings | Coleman, D; Del Quest, A. | 2014 | Social Work in Public Health |
| Population | Identifying and Referring Youths at Risk for Suicide Following Participation in School-Based Gatekeeper Training | Susanne Condron, D; Garraza, L.G; Walrath, C.M; McKeon, R; Goldston, D.B; Heilbron, N.S. | 2014 | Suicide & Life-Threatening Behavior |
| Population | Effectiveness of Australian youth suicide prevention initiatives | Page, A; Taylor, R; Gunnell, D; Carter, G; Morrell, S; Martin, G. | 2011 | British Journal of Psychiatry |
| Population | The short-term effectiveness of a suicide prevention gatekeeper training program in a college setting with residence life advisers | Tompkins, T.L; Witt, J. | 2009 | Journal of Primary Prevention |
| Population | Telephone counselling for adolescent suicide prevention: changes in suicidality and mental state from beginning to end of a counselling session | King, R; Nurcombe, B; Bickman, L; Hides, L; Reid, W. | 2003 | Suicide & Life-Threatening Behavior |
| Population | Many helping hearts: an evaluation of peer gatekeeper training in suicide risk assessment | Stuart, C; Waalen, J.K; Haelstromm, E. | 2010 | Death Studies |
| Population | A consumer's perspective of a suicide intervention programme | Aoun, S; Johnson, L. | 2001 | Australian New Zealand Journal of Mental Health Nursing |
| Population | Intervention styles with suicidal callers at two suicide prevention centers | Daigle, M.S; Mishara, B.L. | 1995 | Suicide & Life-Threatening Behavior |
| Population | Effects of different telephone intervention styles with suicidal callers at two suicide prevention centers: an empirical investigation | Mishara, B.L; Daigle, M.S. | 1997 | American Journal of Community Psychology |
| Population | The decline of youth suicidal behavior in an urban, multicultural public school system following the introduction of a suicide prevention and intervention program | Zenere, F.J 3rd; Lazarus, P.J. | 2011 | Suicide & Life-Threatening Behavior |
| Population | Impact of different interventions on preventing suicide and suicide attempt among children and adolescents in the United States: a microsimulation model study | Zhang, C; Zafari, Z; Slejko, J.F; Camelo Castillo, W; Reeves, G.M; dosReis, S. | 2023 | Frontiers in Psychiatry |
| Population | Long-term impact of the Garrett Lee Smith Youth Suicide Prevention Program on youth suicide mortality, 2006-2015 | Godoy Garraza, L; Kuiper, N; Goldston, D; McKeon, R; Walrath, C. | 2019 | Journal of Child Psychology and Psychiatry and Allied Disciplines |
| Population | Education And Prevention: Strategies To Prevent Adolescent Suicide | Gómez-Delgado, G; Suárez, A.A.G; Núñez, R.P. | 2022 | Journal of Pharmaceutical Negative Results |
| Population | A Community-Based Response to a Suicide Cluster: A Hong Kong Experience | Lai, C.C.S; Law, Y.W; Shum, A.K.Y; Ip, F.W.L; Yip, P.S.F. | 2020 | Crisis |
| Population | Don't Invite Everyone! Training Variables Impacting the Effectiveness of QPR Trainings | Peterson, A.L; Monahan, M.F; Bender, A.M; Gryglewicz, K; Karver, M.S. | 2021 | Administration and Policy in Mental Health and Mental Health Services Research |
| Population | Effectiveness of a school-based mental health education program in an impoverished urban area of Peru | Kim, H.Y; Nam, E.W; Jin, K.N; So, A.Y. | 2020 | Global Health Promotion |
| Population | Culturally informed gatekeeper training for youth suicide prevention in Guyana: A pilot examination | Persaud, S; Rosenthal, L; Arora, P.G. | 2019 | School Psychology International |
| Population | Effect of the Garrett Lee Smith memorial suicide prevention program on suicide attempts among youths | Garraza, L.G; Walrath, C; Goldston, D.B; Reid, H; McKeon, R. | 2015 | JAMA Psychiatry |
| Population | Impact of the Garrett Lee Smith youth suicide prevention program on suicide mortality | Walrath, C; Garraza, L.G; Reid, H; Goldston, D.B; McKeon, R. | 2015 | American Journal of Public Health |
| Outcomes | Decreases in suicide deaths and attempts linked to the white mountain apache suicide surveillance and prevention system, 2001-2012 | Cwik, M.F; Tingey, L; Maschino, A; Goklish, N; Larzelere-Hinton, F; Walkup, J; Barlow, A. | 2016 | American Journal of Public Health |
| Outcomes | The Garrett Lee Smith memorial suicide prevention program | Goldston, D.B; Walrath, C.M; McKeon, R; Puddy, R.W; Lubell, K.M; Potter, L.B.; Rodi, M.S. | 2010 | Suicide and Life-Threatening Behavior |
