# Supplementary material for: Effectiveness of Suicide Prevention Programmes Among Adolescents and Sociocultural Adaptation of Programmes: A Systematic Review
Source: Int J Ment Health Nurs. 2025 Apr 10;34(2):e70038. doi: 10.1111/inm.70038 (PMC11984072; doi:10.1111/inm.70038)
Supplement: Supplementary file 4 — Appendix S4. [file INM-34-0-s002.docx]

**Appendix 4- Further details of studies**

| **Author, Year, Country** | **Aims** | **Study Population** | **Number of schools and participants** | **Retention Rate** | **Gender of participants** | **Follow- Up** |
| --- | --- | --- | --- | --- | --- | --- |
| Bockhoff K, 2023, Germany | To provide training on psycho-educational program for students and to evaluate the benefits on suicide prevention | Age Range: Adolescents from grade 8-10 | School: 12 Participants: 200 | 99 out of 200 | Male: 21% Female: 79% | Post test was done as a follow-up. Total 2 times, post-test and follow-up test after 3 months |
| Calear AL, 2021, Australia | To determine the effectiveness of the SoS program in increasing positive help-seeking intentions for suicide among adolescents | Male Adolescents age of 16-18 years from grade 11-12 | School: 10 Participants: 594 | NA | Only male | Total 2 post-tests was done as follow-ups. First: 1-2 weeks after and second: 6-12 weeks after intervention |
| Calear, AL, 2022 Australia | To evaluate the effectiveness of the school-based Sources of Strength (SOS) program in increasing help-seeking intentions and behaviours among adolescents | 11-17 years (M =13.3 years (SD = 1.2) Grade 7-11 | School: 13 (7 intervention; 6 wait-list control) Participants: 1633 | 1428 completed pre-intervention survey 1125 completed post intervention survey 1048 completed 6 months follow up, 786 completed 18 months follow-up (waitlist control had approximately twice the attrition rate than intervention) | Male: 36.1% in intervention, 48.9% in control group. Female: overall 58% (63% in intervention, 50.4% in control). Others: 0.9% in intervention, 0.7% in control group (n=12) | Post-intervention and at 6 months and 18 months |
| McGillivray, 2021, Australia | To examine the impact of YAM on suicidal ideation, self-reported suicide attempt, suicide literacy, suicide stigma and hep-seeking in Australian secondary school students | 13 to 16 years mean age 14.4 years | School: 18 Participants: 556, 327 in 3 months follow-up, 327 in six months follow-up, 203 completed all the 3 surveys | 203 out of 556 | Male: 43.4% Female: 56.6% (Orientation Heterosexual (506) LGTBW+: 42) | Total 2 post-tests as follow-up. One was 3 months and other 6 months. |
| Ciffone J, 2007, USA | To evaluate the effectiveness of curriculum based high school program in changing attitudes about suicide and its effectiveness while delivering by the different individuals | 10th grade | School: 2 Participants: 421 | NA | Male: 47% Female: 53% Others: | One post-test as follow up after 21 days |
| Cigularov K, 2008, USA | To evaluate the effectiveness of RAPP in knowledge of suicide, attitudes towards suicide and its prevention and identification and help suicidal peer and self-efficacy in seeking and offering help for suicidal thoughts | Knowledge sample mean age 15.3, Attitude samples 14.9 years and self-efficacy group 15.3 years. | School: 7 Participants: 779 | 669 out of 779 | Male: 50% in Knowledge, 52% attitude and 51% in self-efficacy group. Female: 50% in Knowledge, 48% in attitude and 49% in self-efficacy samples | Immediately after the program |
| Portzky G, 2006, Belgium | To examine the effectiveness of psycho-educational program on knowledge, attitudes, coping skills for suicidal feelings | 14 to 18 years, mean age: 15.6 years | School: 10 Participants: 172 | 94% | Male: 37.3% Female: 62.7% Others: | One post-test as follow-up two months after the intervention |
| Kalafat J, 1994, USA | To assess the efficacy of a high school suicide curriculum on youth suicidal behaviour, knowledge, and attitude towards suicide | 10th grade | School: 2 Participants: 253 | NA | Male: 57% Female: NA Others: NA | One post-test as a follow-up after 3 weeks of intervention |
| Hart LM, 2020, Australia | To assess the effectiveness of teen Mental Health First Aid (tMHFA) in improving peer support for adolescents at risk for suicide | 15 to 17 years (M=15.97, SD 0.52), Year 10 | School: 4 Participants: 1605 | 70% n=1116 in first follow-up and 66% n=894 in 12 months follow up | Male:  Female: 37.23% Others: | Total 2 post-test as follow up immediately and 12 months later |
| Schilling EA, 2014, USA | To evaluate the effectiveness of SOS program in high military impact middle schools in reducing suicidal behaviour, increasing knowledge on suicide, and improving attitudes towards help-seeking or offering help | grade 5 to 8 | School: 8 Participants: 470 | 94% n=419 | Male: 47.4% Female: 52.6% | One post-test as a follow-up after 3 months |
| Schilling EA, 2016 USA | To evaluate the effects of Signs of Suicide (SOS) prevention program on self-reported suicide attempts and suicide behaviours of high school students | Grade 9 | School: 16 Participants: 1302 (pre-test); 1268 (post-test) | 1052 completed both pre- and post-test | Male: 58.3% Female: 41.7% | One post-test as follow-up in 3 months after the program |
| Pickering TA, 2022, USA | To assess the effectiveness of the SOS program in reducing suicidal ideation, suicide attempts, knowledge, and attitudes towards suicide among ethnically diverse adolescents while delivering the program by peer leaders | Grade 9-12 Mean 15.7 SD (0.19) | School: 20 Participants: 5746  Peer leader: 459 | 4026 out of 5746 | Male: mean 5.1% SD (3.44%) Female:  Others: | One post-test in 3 months |
| Klimes-Dougan B, 2009, USA | To investigate the possible benefits and untoward effects of suicide prevention public service announcements for adolescents on depression, suicidal behaviours, and coping | Grade 10-12  Mean age= 15.24 SD 0.89 | School: 3 Participants: 426 | NA | Male:  Female: 56% Others: | Immediately after the program (on the basis of depression score participants were categorised into high risk and low risk group and compared |
| Aseltine RH, 2007, USA | To investigate the effectiveness of SOS prevention program in reducing suicidal behaviour, increasing knowledge and improving attitude | Grade 9 | School: 8 Participants: 4133 | Response rate 92% | Male: Hartford 48.7%, Columbus 51.6%, Massachusetts 52.5% Female: Hartford: 51.3%, Columbus 48.4%, Massachusettes 57.5% | one post-test 3 months after the program |
| Aseltine RH, 2004 USA | To investigate the effectiveness of SOS prevention program in reducing suicidal behaviour, increasing knowledge and improving attitude | Grade 9 | School: 5 Participants: 2100 | Response rate 93% | Male: Hartford, 47% Columbus 52% Female: Hartford 53% and Columbus 48% | 3 months after |
| Petrova M, 2015, USA | To investigate the impact of peer-delivered positive-themed suicide prevention message in reducing suicidal behaviours, in identification of trusted adults and attitude of students towards suicide | Grade 9-12 | School: 4 Participants: 833 | 706 responded response rate 84.8% | Male: Female: 49.2% Others: NA | Immediately after the program |
| Hooven C, 2010, USA | To evaluate the long-term effectiveness of promoting CARE suicide prevention program in suicidal risk behaviours of adolescents | Grade 9-12 (mean age 15.9) | School: 20 Participants: 615 | NA | Male: NA  Female: 61%  Others:NA | 7 months after intervention and 2.5 to 8 years when the person becomes young adult |
| Hooven C, 2012, USA | To evaluate the effectiveness of augmenting a youth suicide preventive intervention with a brief home-based parent program in reducing the suicidal risk | 14 to 19 years M=16 years | School: 20 Participants: 2160 among them 615 were eligible | 98% in T1 and 87 % in 15 months | Male: NA  Female: 60% Others:NA | Total 4 baseline-1 month, 2.5 month 9 and 15 months |
| Eggert LL, 2002, USA | To evaluate the efficacy of C-CAST and CAST in reducing the suicidal risk as compared to usual care | 14 to 19 years | School: 7 Participants: 341 | C-Care Retention 89% and 80% in CAST | Male: NA Female: 56% Others:NA | Total 3 baseline-4 weeks, 10 weeks and 9 months |
| Klim-Conforti P, 2021, Canada | To evaluate the impact of Harry Potter-based mental health literacy curriculum on suicidality | 11 to 14 years Grade 7 to 8 | School: 15 Participants: 603 | 430 out of 603 | Male: 164 Female: 265 Others: 1 non-binary | 3 months |
| Wasserman D, 2015 10 European Union Countries | To investigate the efficacy of school-based preventive interventions and compare the effectiveness between QPR, ProfScreen and YAM for suicidal behaviours | 14-16 years M=14.8 years (SD= 0.8) | School: 168 (10 European countries) Participants: 11110  2721 (YAM) 2764 (ProfScreen) 2692 (QPR) 2933 (Control) | 1977 (QPR) 1991 (YAM) 1992 (Prof Screen) 2261 (Control) | Male: NA  Female: 59% Others: NA | 3 months and 12 months after |
| Wyman PA, 2010 USA | To examine the effectiveness of the SOS program in enhancing protective factors among peer leaders and reducing suicide among high school students | Intervention Mean age= 15.7, SD 1.17 and Control Mean age 16.1, SD 1.12 | School: 18 Participants: 16961 (9666 Intervention, 7295 control) | NA | Male: Ranged 23.7-50% to  Female: Ranged from 50-76.3%  across the different groups | One post-test after 4 months |
| Thompson EA, 2000 USA | To assess the effectiveness of Personal Growth Class suicide prevention program in reducing suicidal risk behaviours | Grades 9-12 mean age PGC I: 16.19 SD 0.92  PGC II M=15.82 SD 1.11  PGC III M=15.57 SD 1.01 | School: 5 Participants: 106 | Group I-75% Group II- 87.5% Group III-71.4% | Male: 41.7% in Group I, 37.1% in PGC II and 45.7% in PGC III Female:  Others: | Two post-tests 5 months and 10 months |
| Thompson EA, 2001 USA | To test the efficacy of two interventions for reducing suicidal risk behaviour and related risk factors and for enhancing protective factors | 14 to 19 years | School: 7 Participants: 460 C-Care-150, CAST-155, Control: 155 | 86% for C-CARE, 93% for CAST and 90% control | Male: NA  Female: 52% Others:NA | Total 3 post-test, 4-weeks after baseline, 10 weeks after baseline coinciding with CAST training completion and 9 months after baseline |
| Vieland V, 1991 USA | To assess the impact of curriculum-based suicide prevention program on suicide and help-seeking 18 months after delivery | Mean age 15.8 SD 0.64 | School: 6 Participants: 381 | NA | Male: 184 Female: 197, 55% in experimental and 49% control | NA |
| Orbach I, 1993, Israel | To examine the effectiveness of an experiential suicide prevention program in reducing the suicidal tendency and improving coping | Junior high school | School: 6 Participants: 393 | 39 dropped | Male: 177 Female: 216 | 1 to 3 weeks after |
| Silverstone PH, 2015, Canada | Piloting of a novel school-based approach to reduce depression and suicidality in youth | Adolescents age of 11 to 18 years, Grade 6-12 | School: 5 Participants: 3244 | 2790 | Male: 1676 Female: 1568 | One follow up in 12 weeks |
| Silverstone PH, 2017, Canada | 1)To reduce suicidal thinking in pre-teens, adolescents, and youth students, aged 11–18 in middle schools (Grades 6–8) and high SCHOOLS (Grades 9–12). 2) To reduce depression and anxiety | Adolescents age of 11 to 18 years | School: 5 Participants: 3244 | 1884 | Male: 32% Female: 34%  Others:34% declined to report | 15 months follow up of previous study |
| Paschall MJ, 2018, USA | To assess the decrease in the likelihood of depressive episodes and suicide risk among adolescents with the increasing availability of mental health services at school-based health centers in Oregon public schools. | Adolescents Grade 8-11 | School: 168 Participants: 17307 | NA | Male: NA Female: 51.6% Others: NA | NA |
| Wasserman C, 2012, 11 European countries | Generating recommendations and enhancing the future potential of such suicide prevention strategies | Adolescents mean 14.9 years, SD 0.9 | School: 179 schools, 11 European countries Participants: 12395 | 3016 participated in the awareness (55.2% female and 44.8% male) | Male: 5529 Female: 6700 Others: (67 missed to report) | NA |
| Antonio MCK, 2020, USA | To evaluate the impacts of participating in HCCI for youth suicide prevention | Adolescent leaders age of 13-18 years | School: NA Participants: 17 | NA | Male: Majority Female: NA  Others: NA | HCCI was implemented 4 years back, and focus group discussion was conducted at this time |
| White J, 2010, Canada | To illuminate the uncertainties, complexities, and fresh possibilities of youth suicide prevention education | Adolescents from grade 8-12 (13-15 years) | School: 1 Participants: 6 | NA | NA | Interviewed at least twice at the start of the program and at the end of the program |
| Orlins ER. 2023, USA | To evaluate the effectiveness of LifeAct High School suicide prevention program in knowledge and help-seeking intentions | 13-18 years | School: 10 Participants: 1313 | 1299 | Male: 49.7%  Female: 47.0%  Others:3.3% | Two post-tests as follow ups 2 weeks and 10 weeks after the intervention |
| Orlins, ER, 2023 USA | To explore the perception of suicide among teenagers, to inform existing and future suicide prevention programs and to collect feedback on LifeAct High School program | 9th grade, 13-18 years | School: 1 Participants: 14 | NA | Male: 35.7% Female: 57.1%  Others: 7.1% | NA |
| Langdon SE, 2016 USA | To assess the effectiveness of Lumbee Rite of Passage (LROP) suicide prevention model among American Indian youth | 11-18 years | School: NA Participants: 16 for focus group and 22 for the program | 17 completed the quantitative study | NA | 6 months |
| Le TN, 2015, USA | To investigate the effectiveness of culturally adapted mindfulness-based prevention intervention in preventing suicide of Native American Youth | 15-20 years | School: 2 Participants: 8 | NA | Male: 5  Female: NA  Others: NA | On completion |
| Kinchin I, 2019 Australia | To evaluate SafeTALK program on suicide-related outcomes (Knowledge, attitude, help-seeking and helping others) | 15-16 years | School: 1 Participants: 28 | 22 participants in Time 2 and 12 in Time 3 of data collection. | Male: NA  Female: 82.1%  Others: NA | Two post-tests one is on completion of the program and other was 4 weeks after the program |
| Shaffer D, 1991, USA | To study the effectiveness of suicide curriculum in increasing knowledge, students’ reaction to the program | Grade 9- 10, Mean age was 14.7 for experimental and 14.9 for control group | School: 11 Participants: 2240 | Average 82% in intervention and 98% in control group | Male:  Female: 52% Others: | One post-test after one month of pretest |
| Wise E, 2023, USA | To assess the efficacy of ECPR in improving adolescents' confidence in ability to respond to a peer crisis and ability to identify adequate crisis intervention skills | 14- 18 years mean 15.61, SD=1.37 in experimental and 16.70 SD=1.02 in control group | School: NA Participants: 287 experimental and 220 in control group | NA | Male: 29.4% Female: 68.5% Others: 0.7 in experimental group  Male: 21.4%, Female: 73.6% and 5% transgender in control group | On completion of the intervention |
| Aseltine RH, 2003 USA | To evaluate the efficacy of the SOS program on help-seeking for suicidal behaviour | High school | School: 92 Participants: NA | 74% schools completed the program | NA | One post-test 30 days after the program |
| Bailey E, 2017 Australia | To evaluate efficacy of the SafeTALK for secondary school students on suicidal ideation, knowledge of suicide, confidence and willingness to talk about suicide and offer help, help-seeking and distress | 16-18 years, Grade 11-12; Mage=16.7 years | School: 3 Participants: 335 | Consent rate 61%, 84.9% in pretest, 80.3% on completion and 65.8% in 4 weeks follow up | Male: 53.49% Female: NA  Others:NA | Total 2 post-tests immediately after and 4-week follow up |
| White LL. 2012, USA | To measure the effectiveness of the Erika's Lighthouse Understanding Teen Depression program in changing adolescents' self-reported attitude toward suicide | High-school adolescents | School: 1 Participants: 331 | 306 | NA | Following day of the program |
| Strunk CM, 2014, USA | To determine the impact of Surviving the Teens ® Program in help-seeking behaviour among troubled high risk for suicide teens | 13-18 years | School: 9 Participants: 966 in experimental and 566 in control group | NA | Male: 43.9% in experimental and 50.5% in control group Female: 56.1% in experimental and 49.5% in control group Others: | One post-test 4 days after the program |
| Pasco S, 2012, USA | To evaluate the efficacy of gatekeepers training in improving suicide related knowledge and skills | Mean age 19 More than 75% were below 19 years | School: NA Participants: 85 | 65 | Male: 43.1% Female:  Others: 53.8% | One follow up on completion |
| Freedenthal S, 2010 USA | To assess the efficacy of yellow ribbon program in help-seeking behaviour of adolescents | Grade 9-12 mean age 15.8, SD 1.2 | School: 2 Participants: 783 | 24.1% in experimental group completed pre-test (n=210) survey and 69.6% of retention rate in post-test (n=146) | Male: 40% in experimental 52% in control group  Female: 60% in experimental 48% in control group.  Others: | One post-test in between 6 to 8 months of program implementation |
| Ciffone J, 1993 USA | To assess the effectiveness of a suicide prevention program conducted in a sophomore level health class | High-school adolescents | School: 3 Participants: 203 in intervention and 121 in control group | NA | Male: 119 in experimental and 53 in control groups Female: 84 in experimental and 68 in control group | One follow up 30 days after |
| Nelson FL, 1987 USA | To assess the effectiveness of youth suicide prevention school program on knowledge and attitude towards suicide | Grades 9-12, mean age 15.5 years | School: 8 Participants: 370 | NA | Almost equal proportion of male and female | Following the program |
| Ogawa S, 2022 Japan | To assess the effectiveness of SOS output education in reducing suicidal risk and improving help-seeking | 14 years | School: 1 Participants: 188 | 90.4% in some response and 77.1% other response | NA | Two post-tests one was 3 weeks after the pretest for both groups, one received intervention and other did not. Then second post-test was done 7 weeks after the baseline at this moment both groups had received the intervention |
| Robinson J, 2016, Australia | To test the effects of specifically designed eight-module internet-based program on suicidal ideation among secondary students | 14-18 years mean age 15.6 | School: 11 Participants: 32 | 21 | Male: NA  Female: 81% Others:NA | On completion |
| King KA, 2010 USA | To examine the effects of surviving the Teens® on students' suicidal ideation and suicidal behaviour, self-efficacy regarding suicidal prevention and intervention, and help-seeking behaviour | Grades 9-12, 14-18 years, M = 14.1, SD = .785 | School: NA Participants: 1030 | 89.2% on completion and 40.4% in 3 months | Male: 43.9% Female: 56.1% Others: | Two post-tests on completion and 3 months after |
| Walker RL, 2009, USA | To assess the effects of LifeSavers program on suicide attitudes and knowledge of youth | Grade 9-12 | School: 8 Participants: 63 | NA | Male: 22.2% Female: 77.8% Others: | At the end of training |
| Flynn A, 2016 USA | To evaluate the self-reported changes in knowledge and comfort in communicating suicide following Yellow Ribbon training | 11- 18 years | School: 8 Participants: 3257 | 2857 | Male: Female: 51% Others: | After the program |
| Nasution RA, 2019 Indonesia | To assess the effects of CBT and peer leadership on suicidal ideation of adolescents | Grade 11 | School: 2 Participants: 86 | 100% | Male: 30.2% Female: 69.8% Others: | On completion |
| Baggio, S, 2022 Switzerland | To evaluate the efficacy of a brief universal suicide prevention program on knowledge of suicide, coping skills and help-seeking | Mean age 15.28 years | School: 2 Participants: 373 | 80.20% | Male: 44.1% Female: 55.8% Others: | One post-test after a month |
